# Aromatic inhibitors derived from ammonia-pretreated lignocellulose hinder bacterial ethanologenesis by activating regulatory circuits controlling inhibitor efflux and detoxification

**DOI:** 10.3389/fmicb.2014.00402

**Published:** 2014-08-13

**Authors:** David H. Keating, Yaoping Zhang, Irene M. Ong, Sean McIlwain, Eduardo H. Morales, Jeffrey A. Grass, Mary Tremaine, William Bothfeld, Alan Higbee, Arne Ulbrich, Allison J. Balloon, Michael S. Westphall, Josh Aldrich, Mary S. Lipton, Joonhoon Kim, Oleg V. Moskvin, Yury V. Bukhman, Joshua J. Coon, Patricia J. Kiley, Donna M. Bates, Robert Landick

**Affiliations:** ^1^Great Lakes Bioenergy Research Center, University of Wisconsin-Madison Madison, WI, USA; ^2^Department of Biomolecular Chemistry, University of Wisconsin-Madison Madison, WI, USA; ^3^Department of Biochemistry, University of Wisconsin-Madison Madison, WI, USA; ^4^Department of Chemistry, University of Wisconsin-Madison Madison, WI, USA; ^5^Pacific Northwest National Laboratory Richland, WA, USA; ^6^Department of Chemical and Biological Engineering, University of Wisconsin-Madison Madison, WI, USA; ^7^Department of Bacteriology, University of Wisconsin-Madison Madison, WI, USA

**Keywords:** *Escherichia coli*, lignocellulosic hydrolysate, aromatic inhibitors, transcriptomics, RNAseq, proteomics, ethanol, biofuels

## Abstract

Efficient microbial conversion of lignocellulosic hydrolysates to biofuels is a key barrier to the economically viable deployment of lignocellulosic biofuels. A chief contributor to this barrier is the impact on microbial processes and energy metabolism of lignocellulose-derived inhibitors, including phenolic carboxylates, phenolic amides (for ammonia-pretreated biomass), phenolic aldehydes, and furfurals. To understand the bacterial pathways induced by inhibitors present in ammonia-pretreated biomass hydrolysates, which are less well studied than acid-pretreated biomass hydrolysates, we developed and exploited synthetic mimics of ammonia-pretreated corn stover hydrolysate (ACSH). To determine regulatory responses to the inhibitors normally present in ACSH, we measured transcript and protein levels in an *Escherichia coli* ethanologen using RNA-seq and quantitative proteomics during fermentation to ethanol of synthetic hydrolysates containing or lacking the inhibitors. Our study identified four major regulators mediating these responses, the MarA/SoxS/Rob network, AaeR, FrmR, and YqhC. Induction of these regulons was correlated with a reduced rate of ethanol production, buildup of pyruvate, depletion of ATP and NAD(P)H, and an inhibition of xylose conversion. The aromatic aldehyde inhibitor 5-hydroxymethylfurfural appeared to be reduced to its alcohol form by the ethanologen during fermentation, whereas phenolic acid and amide inhibitors were not metabolized. Together, our findings establish that the major regulatory responses to lignocellulose-derived inhibitors are mediated by transcriptional rather than translational regulators, suggest that energy consumed for inhibitor efflux and detoxification may limit biofuel production, and identify a network of regulators for future synthetic biology efforts.

## Introduction

Elucidation of metabolic and regulatory barriers in microbial conversion of lignocellulosic sugars to ethanol is crucial for both the immediate goal of economical cellulosic ethanol and for the long-term development of next-generation biofuels and sustainable chemicals from renewable biomass. Efficient conversion of lignocellulose (LC) hydrolysates is limited by multiple factors (Mills et al., 2009; Lau and Dale, 2010), including high osmolarity (Underwood et al., 2004; Purvis et al., 2005; Miller and Ingram, 2007), toxicity of the conversion products (Ingram and Buttke, 1984), and inhibitors of microbial metabolism and growth generated during the deconstruction of LC (Zaldivar et al., 1999; Wang et al., 2011a; Tang et al., submitted). Understanding and overcoming the barriers created by LC-derived inhibitors presents significant challenges as their composition can vary depending on the biomass source of LC, the methods used to deconstruct the LC, and the diverse metabolic and regulatory responses of microbes to inhibitors (Klinke et al., 2004; Liu, 2011). Synergy among the inhibitors, the high osmolarity inherent to hydrolysates, and toxicity of conversion products (e.g., ethanol) are additional factors that contribute to the complex molecular landscape of lignocellulosic hydrolysates (Klinke et al., 2004; Liu, 2011; Piotrowski et al., 2014).

Release of sugars from LC typically requires either acidic or alkaline treatment of biomass prior to or coupled with chemical or enzymatic hydrolysis (Chundawat et al., 2011). Acidic treatments generate significant microbial inhibitors by condensation reactions of sugars (e.g., furfural and 5-hydroxymethylfurfural). Microbes typically detoxify these aldehydes by reduction or oxidation to less toxic alcohols or acids (Booth et al., 2003; Herring and Blattner, 2004; Marx et al., 2004; Jarboe, 2011), but these conversions also directly or indirectly consume energy that otherwise would be available for biofuel synthesis (Miller et al., 2009a,b) The impact of these inhibitors is especially significant for C5 sugars like xylose whose catabolism provide slightly less cellular energy (Lawford and Rousseau, 1995), and can be partially ameliorated by replacing NADPH-consuming enzymes with NADH-consuming enzymes (Wang et al., 2013).

Alkaline treatments, for instance with ammonia, are potentially advantageous in generating fewer toxic aldehydes, but the spectrum of inhibitors generated by alkaline treatments is less well characterized and their effects on microbial metabolism are less well understood. We have developed an approach to elucidate the metabolic and regulatory barriers to microbial conversion in LC hydrolysates using ammonia fiber expansion (AFEX) of corn stover, enzymatic hydrolysis, and a model ethanologen (GLBRCE1) engineered from the well-studied bacterium *E. coli* K-12 (Schwalbach et al., 2012). Our strategy is to compare anaerobic metabolic and regulatory responses of the ethanologen in authentic AFEX-pretreated corn stover hydrolysate (ACSH) to responses to synthetic hydrolysates (SynHs) designed to mimic ACSH with a chemically defined medium. GLBRCE1 metabolizes ACSH in exponential, transition, and stationary phases but, unlike growth in traditional rich media (Sezonov et al., 2007), GLBRCE1 enters stationary phase (ceases growth) long before depletion of available glucose but coincident with exhaustion of amino acid sources of organic nitrogen (Schwalbach et al., 2012). The growth-arrested cells remain metabolically active and convert the remaining glucose, but not xylose, into ethanol (Schwalbach et al., 2012).

Our first version of SynH (SynH1) matched ACSH for levels of glucose, xylose, amino acids, and some inorganics, overall osmolality, and the amino-acid-dependent growth arrest of GLBRCE1 (Schwalbach et al., 2012). However, gene expression profiling revealed that SynH1 cells experienced significant osmotic stress relative to ACSH cells, whereas ACSH cells exhibited elevated expression of efflux pumps, notably of *aaeAB* that acts on aromatic carboxylates (Van Dyk et al., 2004), relative to SynH1 cells (Schwalbach et al., 2012). Osmolytes found in ACSH (betaine, choline, and carnitine) likely explained the lower osmotic stress, whereas phenolic carboxylates derived from LC (e.g., coumarate and ferulate) likely explained efflux pump induction possibly *via* the AaeR and MarA/SoxS/Rob regulons known to be induced by phenolic carboxylates (Sulavik et al., 1995; Dalrymple and Swadling, 1997). We also observed elevated expression of *psp, ibp*, and *srl* genes associated with ethanol stress at ethanol concentrations three-fold lower than previously reported to induce expression (Yomano et al., 1998; Goodarzi et al., 2010) and thus consistent with a synergistic stress response with the LC-derived inhibitors. These findings led us to hypothesize that the collective effects of osmotic, ethanol, and LC-derived inhibitor stresses created an increased need for ATP and reducing equivalents that was partially offset in early growth phase by catabolism of amino acids, as N and possibly S sources. However, as these amino acids are depleted, cells transition to stationary phase where they continue to catabolize glucose for maintenance ATP and NAD(P)H but are unable to generate sufficient energy for cell growth or efficient xylose catabolism.

To test this hypothesis, we developed a new SynH formulation (SynH2) that faithfully replicates the physiological responses in ACSH and the effects of LC-derived inhibitors. Using SynH2 with and without the LC-derived inhibitors, we generated and analyzed metabolomic, gene expression, and proteomic data to define the effects of inhibitors on bacterial gene expression and physiology. The analysis allowed identification of key regulators that may provoke stress responses in the presence of LC-derived inhibitors and suggest that coping mechanisms employed by *E. coli* to deal with lignocellulosic stress drains cellular energy, thus limiting xylose conversion.

## Materials and methods

### Reagents

Reagents and chemicals were obtained from Thermo Fisher Scientific (Pittsburgh, Pennsylvania, USA) or Sigma Aldrich Co. (Saint Louis, Missouri, USA) with the following exceptions. 5-hydroxymethyl-2-furancarboxylic acid and 5-(hydroxymethyl)furfuryl alcohol were obtained from Toronto Research Chemicals Inc. (Toronto, Ontario, Canada). Deuterated compounds for HS-SPME-GC/IDMS were obtained from C/D/N Isotopes (Pointe-Claire, Quebec, Canada). D4-acetaldehyde and U^13^C_6_-fructose were obtained from Cambridge Isotope Labs (Andover, Massachusetts, USA).

### Synthesis of feruloyl and coumaroyl amides

Twenty grams of ferulic or coumaric acid were dissolved in 200 ml of 100% ethanol in a 3-neck, 250 ml round-bottom flask equipped with a magnetic stir bar and a drying tube on one of the outside arms. Ten milliliters of acetyl chloride was added and incubated with stirring at room temperature overnight. Ethanol was removed in a rotary evaporator at 40°C under modest vacuum; the syrup re-dissolved in 250 ml 100% ethanol and re-evaporated twice. When the final syrup was reduced to <25 ml, ~6 ml portions were transferred to heavy-wall 25 × 150 mm tubes containing ~30 ml concentrated ammonium hydroxide and sealed with a Teflon-lined cap. The sealed tubes were incubated at 95°C in a heating block covered with a safety shield overnight. The tubes were cooled and then left open in a hood for 4–8 h to allow evaporation of ammonium hydroxide, during which the feruloyl or coumaroyl amide precipitated. The crystallized products were collected under vacuum on a glass filter and washed with 250 ml ice-cold 150 mM ammonium hydroxide. The product was allowed to air dry in a plastic weigh boat in the hood at room temperature for 2–3 days. Purity of the products was analyzed by silica gel TLC developed with 5% methanol in chloroform. Only preparations exceeding 90% purity were used for experiments.

### Preparation of ACSH

ACSH was prepared by one of two methods that differed in whether or not CS was autoclaved prior to enzymatic hydrolysis. Non-autoclaved CS hydrolysate more closely replicates an industrial process, was used by Tang et al. (submitted) for compositional analysis, and was used for some of our fermentation experiments. Autoclaved CS hydrolysate ensures sterility for bacterial fermentations and was used for our compositional analysis and for experiments to generate RNA-seq data. We did not observe a significant difference in GLBRCE1 behavior in non-autoclaved vs. autoclaved CS hydrolysates, although HMF was detectable in the former, but not the latter (**Table 2**). We observed minor variations in growth with CS harvested in different years. For autoclaved CS hydrolysate, AFEX-pretreated CS was mixed with water to 6–10 L final volume at 60 g glucan/L loading (18–22% solids, adjusted for moisture content) and autoclaved for 30–120 min in a 15 L Applikon bioreactor vessel (Schwalbach et al., 2012). For non-autoclaved CS hydrolysate, AFEX pretreated-corn stover was added to the vessel after the water was autoclaved for 30 min. For both, the sample was cooled to ~70°C, adjusted to 10 L volume with water, and pH adjusted with ~30 ml concentrated HCl. Hydrolysis was initiated by adding Novozymes CTec2 to 24 mg/g glucan and HTec2 to 6 mg/g glucan, followed by incubation for 5 days at 50°C with stir speed at 700 rpm. Some older batches of hydrolysate were prepared using Genencor Accellerase, Genencor Accellerase XY, and Multifect pectinase A in place of Novozyme enzymes (Schwalbach et al., 2012). Solids were then removed by centrifugation (8200 × g, 4°C, 10–12 h) and the supernatant was filter-sterilized through 0.5 μm and then 0.2 μm filters. Prior to fermentation, the hydrolysate was adjusted to pH 7.0 using NaOH pellets and filtered again through a 0.2 μm filter to remove precipitates and to ensure sterility.

### Preparation of synthetic hydrolysate (SynH2)

SynH2 (Table 1) was prepared by combining per L final volume of SynH2 the following ingredients. Water (~700 ml) was mixed with 6.25 ml of 1.6 M KPO_4_ buffer, pH 7.2, 20 ml of 1.5 M ammonium sulfate, 20 ml of 2.25 M KCl, 1.25 M NaCl, 20 ml of a 50X amino acid stock giving the final concentrations shown in Table 1 (except tyrosine), 20 ml of 8.75 mM tyrosine dissolved in 50 mM HCl, 50 ml of 1 mM each adenine, guanine, cytosine and uracil dissolved in 10 mM KOH, 10 ml of vitamin stock (1 mM each thiamine, calcium pantothenate, *p*-aminobenzoic acid, *p*-hydroxybenzoic acid, and 2,3-dihydroxybenzoic acid), 1 ml of a 1000X stock of micronutrients (ZnCl_2_, MnCl_2_, CuCl_2_, CoCl_2_, H_3_BO_3_, (NH_4_)_6_Mo_7_O_24_, and FeCl_3_) giving the final concentrations shown in Table 1, 1 ml of 1 M magnesium chloride, 1 ml of 90 mM CaCl_2_, 10 ml of 1 M sodium formate, 10 mM sodium nitrate, and 50 mM sodium succinate, 1 ml of 3 M glycerol, 1 ml of 500 mM lactic acid, 1 ml of 700 mM glycine betaine, 700 mM choline chloride, 200 mM DL-carnitine (osmolytes), 5.61 g acetamide, 2.71 g sodium acetate, 3.3 g sodium pyruvate, 2.94 g sodium citrate, 1.34 g DL-malic acid, 60 g D-glucose, 30 g D-xylose, 5.1 g D-arabinose, 1.48 g D-fructose, 1.15 g D-galactose, and 468 mg D-mannose. After adjusting to pH 7 with 10 N NaOH, the final volume was adjusted to 1 L. This base recipe corresponds to SynH2^−^. To create SynH2, the aromatic inhibitors were added as solids to the base recipe in the following quantities per L SynH2 and stirred until fully dissolved before filter sterilization; 531 mg feruloyl amide, 448 mg coumaroyl amide, 173 mg *p*-coumaric acid, 69 mg ferulic acid, 69 mg hydroxymethylfurfural, 59 mg benzoic acid, 15 mg syringic acid, 14 mg cinnamic acid, 15 mg vanillic acid, 2 mg caffeic acid, 20 mg vanillin, 30 mg syringaldehyde, 24 mg 4-hydroxybenzaldehyde, 3.4 mg 4-hydroxybenzophenone. For some experiments (Figures S3, S4), feruloyl amide, coumaroyl amide, *p*-coumaric acid, ferulic acid, and hydroxymethylfurfural were added at up to twice these concentrations. The medium was filter-sterilized through a 0.2 μm filter.

**Table 1:** **Composition of ACSH, SynH1, SynH2^−^, and SynH2**.

**Media component**	**ACSH**		**SynH1^a^**	**SynH2^−^**	**SynH2**
**CARBOHYDRATES (mM)^a^**					
D-Glucose	343		333	333	333
D-Xylose	205		200	200	200
L-Arabinose^b^	31		–	–	–
D-Arabinose^b^	–^c^		–	34	34
D-Galactose	7.6		–	6.4	6.4
D-Mannose	3.5		–	2.6	2.6
L-Rhamnose	0.9		–	–	–
L-Fucose	0.2		–	–	–
D-Fructose	8.2		–	8.2	8.2
**MISC. COMPOUNDS (mM)^a^**					
Lactate	0.5		–	0.5	0.5
Pyruvate	–		–	30	30
Citrate	–		–	10	10
Nitrate	–		–	0.1	0.1
Formate	11.2		–	10	10
Malate	9.3		–	10	10
Succinate	0.8		–	0.5	0.5
Acetate	36		–	33	33
Acetamide	76		–	95	95
Glycerol	5.5		–	3	3
Glycine betaine	0.7		–	0.7	0.7
Choline	0.7		–	0.7	0.7
Carnitine	0.2		–	0.2	0.2
**SALTS (mM)**					
KH_2_PO_4_	–		22	3.4	3.4
K_2_HPO_4_	–		42	6.6	6.6
KCl	–		45	45	45
NaCl	–		25	25	25
(NH_4_)_2_SO_4_	–		30	30	30
MgCl_2_	–		1	1	1
CaCl_2_	–		0.09	0.09	0.09
**AMINO ACIDS (μM)^a^**					
Alanine	760		700	700	700
Arginine	370		400	400	400
Asparagine	163		200	200	200
Aspartate	379		350	350	350
Cysteine	n.d.^d^		50	50	50
Glutamine	102		100	100	100
Glutamate	459		450	450	450
Glycine	542		400	400	400
Histidine	56		80	80	80
Isoleucine	0		250	250	250
Leucine	460		360	360	360
Lysine	187		200	200	200
Methionine^d^	n.d.		100	100	100
Phenylalanine	189		200	200	200
Proline	214		225	225	225
Serine	272		275	275	275
Threonine^d^	216		225	225	225
Tryptophan	n.d.		50	50	50
Tyrosine	171		175	175	175
Valine	270		225	225	225
**NUCLEOBASES (μM)**					
Adenine	–		100	100	50
Cytosine	–		100	100	50
Uracil	–		100	100	50
Guanine	–		100	100	50
**TRACE COMPONENTS (μM)**					
Thiamine-HCl	–		10	10	10
Pantothenate	–		10	10	10
p-Aminobenzoic acid	–		10	10	10
p-Hydroxybenzoic acid	–		10	10	10
2,3-di-Hydroxybenzoic acid	–		10	10	10
CuCl_2_	–		0.010	0.010	0.010
CoCl_2_·6H_2_O	–		0.025	0.030	0.030
H_3_BO_4_	–		0.400	10	10
(NH_4_)_6_Mo_7_O_2_·4H_2_O	–		0.003	0.003	0.003
FeCl_3_	–		16.6	17	17
ZnCl_2_	–		12	12	12
MnCl_2_·4H_2_O	–		100	100	100
**LC-derived inhibitors (mM)^e,f^**	**w/o auto-claving^g^**	**w/auto-claving**			
Feruloyl amide	5.5	3.5 ± 0.6	–	–	2.75
Coumaroyl amide	5.5	7.1 ± 1.3	–	–	2.75
Hydroxymethylfurfural	1.1	<0.03	–	–	0.55
*p*-Coumaric acid	2.1	1.4 ± 0.3	–	–	1.05
Ferulic acid	0.71	0.091 ± 0.003	–	–	0.355
Benzoic acid	0.48	0.32 ± 0.01	–	–	0.48
Syringic acid	0.08	0.036 ± 0.004	–	–	0.08
Cinnamic acid	0.09	–	–	–	0.09
Vanillic acid	0.09	0.15 ± 0.02	–	–	0.09
Caffeic acid	0.01	0.006 ± 0.001	–	–	0.01
Vanillin	0.132	0.24 ± 0.04	–	–	0.132
Syringaldehyde	0.162	0.017 ± 0.002	–	–	0.162
4-Hydroxybenzeldehyde	0.197	0.15 ± 0.02	–	–	0.197
4-Hydroxyacetophenone	0.025	0.017 ± 0.002	–	–	0.025
Osmolality (mol/kg)	1.16 ± 0.03		0.97	1.17 ± 0.01	1.19 ± 0.01

a *ACSH data are from Schwalbach et al. (2012). Sugar concentrations are averages of HPLC-MS and NMR determinations*.

b *In the SynH2 recipe, D-Arabinose was substituted for the L-Arabinose present in ACSH to avoid AraC-mediated repression of xylose-utilization genes (Desai and Rao, 2010). In other contexts, use of L-Arabinose in SynH2 would be appropriate*.

c *–, not determined in ACSH or not added in SynH*.

d *n.d., not detectable by methods used*.

e *Aromatic compounds detected at less than 20 μM in ACSH are not reported here*.

f *The sets of acids, amides, and aldehydes used for supplemental studies in formulating SynH2 consisted of p-Coumaric acid, Ferulic acid, Benzoic acid, Syringic acid, Cinnamic acid, Vanillic acid, and Caffeic acid (acids), Feruloyl amide and Coumaroyl amide (amides), and HMF, Vanillin, Syringaldehyde, 4-Hydroxybenzaldehyde, and 4-Hydroxyacetophenone (aldehydes) at the concentrations listed for non-autoclaved ACSH or fractions thereof as described in the Supplemental Results*.

g *ACSH Inhibitor concentrations for non-autoclaved CS hydrolysate are from (Tang et al., submitted). Hydrolysate preparations are described in Materials and Methods*.

### Chemical analysis of ACSH

Carbohydrates, ethanol, and short chain acids in ACSH and fermentation media were quantified using HPLC-RID, NMR, and GC-MS as previously described (Schwalbach et al., 2012). ACSH osmolality was measured using a Vapro osmometer 5520 (Wescor Inc., Logan, Utah, USA). The synthetic hydrolysate medium used in these studies (SynH2) was based on a previously described synthetic hydrolysate medium (Schwalbach et al., 2012) that was modified to more closely approximate the composition of ACSH media, particularly with regard to the presence of alternative carbon sources and protective osmolytes. Concentrations of components in the modified SynH2 are described in Table S1.

### Fermentative growth conditions

Cell culture was conducted as described previously (Schwalbach et al., 2012), except fermentations were carried out in 3 L bioreactors (Applikon Biotechnology) containing 2.45 L of ACSH or SynH media, and cultures were diluted into ACSH or SynH with initial OD_600_ at 0.2, grown anaerobically overnight, and then inoculated into bioreactors to a starting OD_600_ of 0.2. For fermentation experiments to determine the effect of osmolytes, it was carried out in 0.5 L Sartorius BIOSTAT Qplus bioreactors (Sartorius Stedium Biotech, Bohemia, NY) containing 0.35 L of SynH2 media in the absence or presence of osmolytes or aromatic inhibitors. Culture density was measured using a Beckman Coulter DU720 in a 1 ml cuvette. Due to the high absorbance of ACSH at 600 nm, cells were diluted 1:10 in water prior to OD_600_ measurement, with diluted ACSH (1:10) as a blank. For SynH, diluted SynH (1:10) was used as a blank.

### RNA-seq gene expression analyses

Samples for RNA-seq were captured and RNA extracted as described previously (Schwalbach et al., 2012). FASTQ formatted sequence files from strand-specific Illumina RNA-Seq reads were aligned to the GLBRCE1 reference genome using Bowtie version 0.12.7 (Langmead et al., 2009) with “*—nofw*” strand-specific parameter and maximal distance between the paired reads of 1000 bp. Nucleotide-level read quality information was used to weight each alignment at subsequent probabilistic expression counting step using the RNA-Seq by Expectation-Maximization (RSEM) version 1.2.4 (Li and Dewey, 2011). Posterior mean estimates of counts and FPKM values were used in the downstream analysis.

The program edgeR v.3.0.2 (Robinson et al., 2010) was used to compute differential expression by using the procedures and steps described in the package documentation in all function calls with median normalization rather than the default TMM procedure. We found that median normalization better adjusted for the particular biases within the dataset. Adjusted *p*-values for multiple hypothesis corrections were used as calculated by edgeR. Pairwise fold-changes and adjusted *p*-values are calculated between media types and within each phase and between phases within each media type.

To catalog the most significant effects, we examined the ratios using several different strategies. In addition to identifying the largest changes in expression of individual genes in SynH2 and ACSH relative to SynH2^−^ (Table S2), we also used gene set enrichment analyses as described by Subramanian et al. (2005) and Varemo et al. (2013). We compiled gene sets for these analyses from pathways, transporters, and regulons documented in Ecocyc (Keseler et al., 2013) and KEGG.

### Proteomic measurements

Thirty-four *Escherichia coli* samples were processed for analysis by mass spectrometry at PNNL. Each sample was typically digested using a global urea digestion (Pasa-Tolic et al., 2004; Smyth, 2004) prior to isobaric labeling with an iTRAQ 4-plex labeling kit, following the manufacturer's directions (ABSciex, Redwood City, CA) (Ross et al., 2004; Bantscheff et al., 2008). Prior to high pH reverse phase fractionation with concatenated pooling (Wang et al., 2011b), the samples were desalted using C18 solid-phase extraction (SPE) (SUPELCO, Bellefonte, PA). All samples were processed with a custom LC system using reversed-phase C18 columns (unpublished variation of Maiolica et al., 2005) and the samples were then analyzed with a Velos Orbitrap mass spectrometer (Thermo Scientific, San Jose, CA) that was equipped with an electrospray ionization (ESI) interface (Kelly et al., 2006).

Raw files were searched against a concatenated *Escherichia coli* K-12 database and contaminant database using MS-GF+ (v9018) with oxidation as a dynamic modification on methionine and 4-plex iTRAQ label as a static modification (Kim et al., 2008). The parent ion mass tolerance was set to 50 ppm. The resulting sequence identifications were filtered down to a 1% false discovery rate using target-decoy approach and MS-GF derived *q*-values. Reporter ion intensities were quantified using the tool MASIC (Monroe et al., 2008). Results were then processed with the MAC (Multiple Analysis Chain) pipeline, an internal tool which aggregates and filters data. Missing reporter ion channel results were retained. Degenerate peptides, i.e., peptides occurring in more than one protein, were filtered out. Proteins with one peptide detected were removed if they were not repeatable across at least two replicates. Redundant peptide identification reporter ions were summed across fractions and median central tendency normalization was applied to account for channel bias. Each 4-plex sample group was normalized using a pooled sample for comparison between groups. The final protein values were obtained by averaging their associated peptide intensity values and varied from ~5000 to 350000. Finally, the protein values were then log_2_ transformed.

All proteins that had missing values in their replicates were removed and the pair-wise protein expression level changes and significance *p*-values between the SynH2 and SynH2^−^ cells at each growth phase were estimated using limma (Smyth, 2004; Smith, 2005), which fits a linear model across the replicates to calculate the fold changes, smooths the standard errors for significance and adjusts the *p*-values via the Benjamini-Hochberg method.

### Comparison of proteomic data to transcriptomic data

Pair-wise RNA expression level changes and significance *p*-values were estimated using the edgeR package as previously discussed. The log2-fold-changes for the Protein and RNA were z-score scaled separately to correct for the difference in dynamic ranges between the protein and RNA measurements.

Significant discrepant Protein/RNA ratios between SynH2 and SynH2^−^ cells were estimated using a two-sample *z*-test and the corresponding *p*-values are adjusted for multiple comparisons using the Benjamini-Hochberg method. All Protein/RNA ratios that are either significant in the RNA or protein ratio (*p* < 0.05) and that significantly disagree (*p* < 0.05) are tabulated in Table S7.

## Measurement of internal metabolite abundances

### Preparation of intracellular extracts

Two ml of cell culture was rapidly removed from bioreactors with a 10 ml sterile syringe and cells captured on Whatman 0.45 um nylon syringe filters (GE Healthcare Bio-Sciences, Pittsburgh, Pennsylvania, USA) as described previously (Schwalbach et al., 2012). To reduce the background associated with metabolites present in ACSH and SynH the cells on the filter were then rapidly washed with 5 ml of M9 medium (Neidhardt et al., 1974) lacking a carbon source. Acetonitrile-methanol-water (40:40:20; 2 ml) containing 0.1% formic acid was then applied to the filters, and the eluate captured in a 15 ml conical tube. The eluate was passed through the cells a second time to ensure complete cell lysis and then flash frozen in a dry ice/ethanol bath.

### Detection/quantification of metabolites

The concentration of internal glycolytic and TCA cycle intermediates were determined using high performance anion exchange chromatography electrospray ionization tandem mass spectrometry (HPAEC-ESI-MS/MS). Reagents and non-labeled reference compounds were from Sigma Aldrich Co.

*HPAEC* was adapted from a previously reported method (Buescher et al., 2010), and was used for determination of pyruvate, citrate, α–ketoglutarate, glucose-6-phosphate, fructose-6-phosphate, fructose-1,6-bis phosphate, phospho(enol)pyruvate, and ATP. Chromatography was carried out on an Agilent 1200 series HPLC comprised of a vacuum degasser, binary pump, and a heated column compartment, and a thermostated autosampler set to maintain 6°C. Mobile Phase A was 0.5 mM NaOH and mobile phase B was 100 mM NaOH. Compounds were separated by a gradient elution of 0.35 mL per minute starting at 10% B, increased to 15% B over 5 min and held at 15% B for 10 min, then increased to 100% B over 12 min and held for 10 min before returning to 10% B to be re-equilibrated for 5 min prior to the next injection. The column temperature was 40°C. The injection volume was 20 μL of intracellular extract or calibrant standard mixture.

## Measurement of aromatic inhibitors in ACSH and SynHd

Samples of ACSH and SynH cultures were prepared by centrifugation as described previously (Schwalbach et al., 2012), and then were subjected to reverse phase HPLC high resolution/accurate mass spectrometry (RP-HPLC-HRAM MS) and headspace solid-phase microextraction gas chromatography-isotope dilution mass spectrometry (HS-SPME/IDMS) analysis.

The majority of phenolic compounds were determined by RP-HPLC-HRAM MS, which was carried out with a MicroAS autosampler (Thermo Scientific) equipped with a chilled sample tray and a Surveyor HPLC pump (Thermo Scientific) coupled to a Q-Exactive hybrid quadrupole/orbitrap mass spectrometer by electrospray ionization. The analytical column was an Ascentis Express column (150 × 2.1 mm × 2.7 μm core-shell particles, Supelco, Bellefonte, PA) protected by a 5 mm C18 precolumn (Phenomenex, Torrance, CA). Mobile phase A was 10 mM formic acid adjusted to pH 3 with ammonium hydroxide and mobile phase B was methanol with 10 mM formic acid and the same volume of ammonium hydroxide as was added to mobile phase A. Compounds were separated by gradient elution. The initial composition was 95% A, which was held for 2 min after injection, then decreased to 40% A over the next 8 min, changed immediately to 5% A and held for 5 min, then changed back to 95% A for a column re-equilibration period of 7 min prior to the next injection. The flow rate was 0.3 mL/min.

The HPLC separation was coupled to the mass spectrometer via a heated electrospray (HESI) source (HESI II Probe, Thermo Scientific). The operating parameters of the source were: spray voltages: +3000, −2500; capillary temperature: 300°C; sheath gas flow: 20 units; auxiliary gas flow: 5 units; HESI probe heater: 300°C. Spectra were acquired with fast polarity switching to obtain positive and negative mode ionization chromatograms in a single analysis. In each mode, a full MS^1^ scan was performed by the Orbitrap analyzer followed by a data dependent MS^2^ scan of the most abundant ion in the MS^1^ scan. The Q-Exactive parameters (both positive and negative modes) were: MS^1^ range 85–500 Th, resolution: 17,500 (FWHM at 400 *m/z*), AGC target: 1e6, maximum ion accumulation time 100ms, S-lens level: 50. Settings for data dependent MS^2^ scans were: isolation width: 1.8 Th, normalized collision energy: 50 units, resolution: 17,500, AGC target: 2e5, maximum ion accumulation time: 50 ms, underfill ratio: 1%, apex trigger: 5–12 s, isotope exclusion enabled, dynamic exclusion: 10 s.

HS-SPME/IDMS was used to quantify acetaldehyde, acetamide, furfural, furfuryl alcohol, HMF, 5-(hydroxymethyl)furfural (HMF), and Bis(hydroxymethyl) furan (“HMF alcohol”/BHMF). Samples were thawed and briefly vortex mixed prior to measuring 500 microliters of sample, 500 microliters of stable isotope labeled internal standard mixture, and ~300 mg of NaCl into a 20 mL screw top headspace and quickly capped with magnetic screwtop cap with 4 mm PTFE backed silicone rubber septum for SPME. Automated SPME sample processing and analysis was carried out using a Pegasus 4D GCxGC-TOF MS (Leco Corp. Saint Joseph, Michigan) with an Agilent 6890A gas chromatograph coupled to the ToF mass analyzer via a heated capillary transfer line, and a Gerster-LEAP combi PAL autosampler and sample preparation system with Twister heated sample agitator fitted with an automated SPME holder containing a gray hub 50/30, 23 ga. Stabiliflex DVB/Carboxen/PDMS SPME fiber (Supelco, Inc.). Chromatof software (Leco, Corp.) V. 4.50.8.0 was used for system control during acquisition and for data processing, calibration and calculation of final concentrations. Sample incubation temperature 95°C, agitation speed 100 rpm, during extraction time, 100 rpm, agitation on 4 s/off 15 s, sample extraction time (SPME fiber exposed to the sample headspace in heated agitator) 20 min, desorb time (SPME fiber inserted in hot GC inlet) 60 min. GC cycle time 40 min. Critical injector positions were determined empirically through trial, error, and careful measurement: vial penetration 11 mm, Injector penetration 54 mm, Injector penetration—needle 40 mm. GC was carried out using a StabilWAX-DA column (Restek Corp, Bellefonte, Pennsylvania, USA) 0.25 mm ID × 30 m, *df* = 0.25 μm; carrier gas He, 1 mL/min; split 5:1; purge flow 3 mL/min; inlet temp 250°C; inlet liner type straight split/splitless deactivated glass 0.75 mm ID; equilibration time 1 min; Oven temperature program: initial temperature 30°C, hold 2 min. Increase to 10°C/min to 250°C, hold 10 min; MS transfer line 250°C. ToF mass spectrometer (unit mass resolution) Acquisition delay 85 s; start mass 10 end mass 500; acquisition 10 spectra/s; electron multiplier delta V 1475 (dependent on QC procedure) source temperature 200°C.

Quantification of organic acids in ACSH was carried out by HPAEC-MS/MS in a similar manner to that described for intracellular metabolites.

## Database submissions and accession numbers

Transcriptomic data (RNA-seq and microarray) have been deposited in NCBI's Gene Expression Omnibus and are accessible through GEO Series accession number GSE58927. Proteomic data can be obtained from the PeptideAtlas database (http://www.peptideatlas.org/PASS/PASS00514).

## Results

### SynH2 recapitulates the growth, sugar consumption, and ethanol production profiles of *E. coli* in ACSH

We first sought to validate a new SynH recipe (SynH2) that would replicate ACSH composition and effects on cells. In addition to protective osmolytes, trace carbohydrates, organic acids, acetamide, and alternative electron donors/acceptors detected in ACSH previously (Schwalbach et al., 2012), new compositional analyses revealed significant quantities of coumarate, coumaroyl amide, ferulate, feruloyl amide, 5-hydroxymethylfurfural (HMF) and nine other aromatic carboxylates or aldehydes in ACSH (Table 1). To formulate a chemically defined ACSH-mimic (SynH2) for use with *E. coli*, we tested combinations of the osmolytes and the LC-derived inhibitors in a base medium composition that included the other missing components (Supplemental Results; Materials and Methods), but substituting D-arabinose for L-arabinose to avoid repression of xylose-utilization genes (Desai and Rao, 2010).

**Figure 1:**
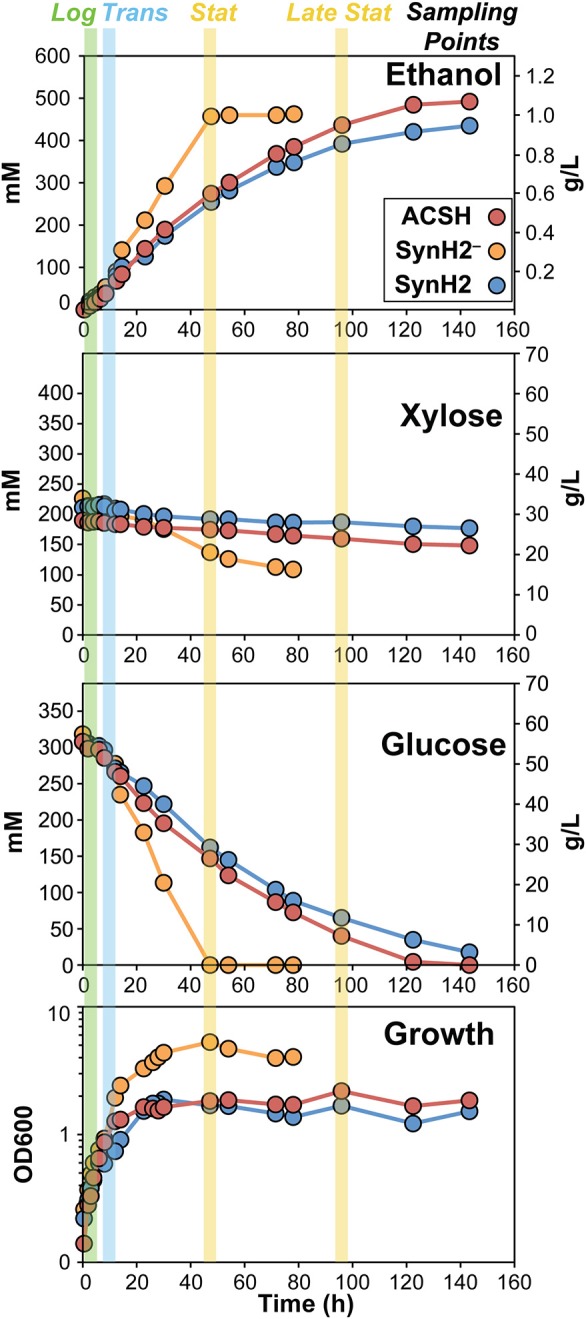
**Growth, sugar utilization, and ethanol production of GLBRCE1 in ACSH, SynH2, and SynH2^−^**. GLBRCE1 was cultured under anaerobic conditions at 37°C in a bioreactor in ACSH, SynH2, or SynH2^−^ (SynH2 lacking aromatic inhibitors; Materials and Methods). Cell density measurements (**bottom panel**), changes in glucose and xylose concentrations in the extracellular medium (**middle panels**), and ethanol concentrations in the vessel (**top panel**) were periodically determined and plotted relative to time. Blue, green, and yellow shaded bars represent points at which samples for metabolite, RNA, and protein analyses were collected during exponential, transition, and stationary phases of growth.

To verify that SynH2 recapitulates the major properties of ACSH and to prepare samples for gene expression and proteomic analyses, we compared growth of the *E. coli* ethanologen in SynH2^−^ (SynH2 lacking aromatic inhibitors), SynH2, and ACSH. For each medium, growth could be divided into exponential, transition, stationary, and late stationary growth phases (Figure 1 and Figure S5). Growth rates of GLBRCE1 in each phase and final cell density were similar for SynH2 and ACSH, with only slight differences, whereas removal of inhibitors (SynH2^−^) significantly increased growth and final cell density (Figure 1 and Figure S5; Table 2). During exponential phase, glucose uptake was similar in all media. As observed previously in ACSH (Schwalbach et al., 2012), cells stopped growth prematurely in both ACSH and SynH, but remained metabolically active and continued glucose assimilation during stationary phase. However, in SynH2^−^, cell growth continued until the glucose was essentially gone (Figure 1 and Figure S5). Thus, cessation of cell growth and entry into the metabolically active stationary phase was caused by the presence of LC-derived inhibitors. In the absence of inhibitors, cells growth ceased when glucose was depleted. In the presence of inhibitors, cells ceased growth when they ran out of organic N and S sources (Schwalbach et al., 2012).

**Table 2:** **Growth, sugar uptake, and ethanol production by GLBRCE1 grown in ACSH and SynH2^−^, and SynH2^a^**.

	**Media**		
	**SynH2^−^**	**SynH2**	**ACSH**
Growth (Exponential) (hr^−1^)^b^	0.13 ± 0.01	0.09 ± 0.02	0.12 ± 0.01
Glucose Rate (Exponential)^b^	4.7 ± 0.5	5.9 ± 1.3	5.6 ± 1.3
Glucose Rate (Transition)^c^	3.2 ± 0.1	2.6 ± 0.4	2.7 ± 0.1
Xylose Rate (Transition)^c^	0.6 ± 0.1	0.5 ± 0.1	0.2 ± 0.1
Glucose Rate (Glu-Stationary)^d^	N/A	1.6 ± 0.2	1.4 ± 0.2
Xylose Rate (Glu-Stationary)^d^	N/A	0.11 ± 0.05	0.11 ± 0.04
Xylose Rate (Xyl-Stationary)^e^	0.19 ± 0.03	0.01 ± 0.01	0.04 ± 0.03
Total Glucose Consumed (mM)	330 ± 20	310 ± 20	300 ± 20
Total Xylose Consumed (mM)	65 ± 30	25 ± 1	25 ± 10
Total Ethanol produced (mM)	540 ± 30	460 ± 60	470 ± 60
Ethanol Yield (%)^f^	70 ± 3	70 ± 6	73 ± 3

a *Each value is from at least three biological replicates in different bioreactors*.

b *Exponential phase is between 4 and 12 h in all media. Unit for glucose uptake rate is mM· OD^−1^_600_ ·h^−1^*.

c *Transition phase is between 12 and 30 h for SynH2-, and between 12 and 23 h for SynH2 and ACSH. Units for glucose and xylose uptake rate are mM · OD^−1^_600_·h^−1^*.

d *Stationary phase when glucose is present (Glu-Stationary) is between 23 and 100 h for SynH2 and ACSH. However, there was no Glu-stationary phase for SynH2^−^ because it remained in transition phase until the glucose was gone*.

e *Stationary phase when glucose is gone (Xyl-Stationary) is between 47 and 78 h for SynH2^−^. The Xyl-Stationary rates for SynH2 and ACSH were measured in follow-up experiments carried out long enough to exhaust glucose in stationary phase*.

f *Calculated from the total ethanol produced and the total glucose and xylose consumed, assuming 2 ethanol per glucose and 1.67 ethanol per xylose*.

After glucose depletion and entry into stationary phase in SynH2^−^, GLBRCE1 consumed xylose (up to ~50% by the time the experiments were terminated 80–100 h; Figure 1 and Figure S5; Table 2). However, little xylose consumption occurred in the presence of inhibitors or in ACSH, presumably in part because glucose conversion continued during stationary phase to near the end of the experiment. However, even in experiments that exhausted glucose in stationary phase, SynH2 cells and ACSH cells exhibited little or no xylose conversion (Table 2). GLBRCE1 generated slightly more ethanol in SynH2^−^ than in SynH2 or ACSH, consistent with greater sugar consumption, but also generated ethanol much faster than in the inhibitor-containing media (Figure 1 and Figure S5; Table 2). We conclude that LC-derived inhibitors present in SynH2 and in ACSH cause *E. coli* cells to cease growth before glucose was consumed, decreased the rate of ethanol production, and to lesser extent decreased final amounts of ethanol produced.

### GLBRCE1 gene expression patterns are similar in SynH2 and ACSH

**Figure 2:**
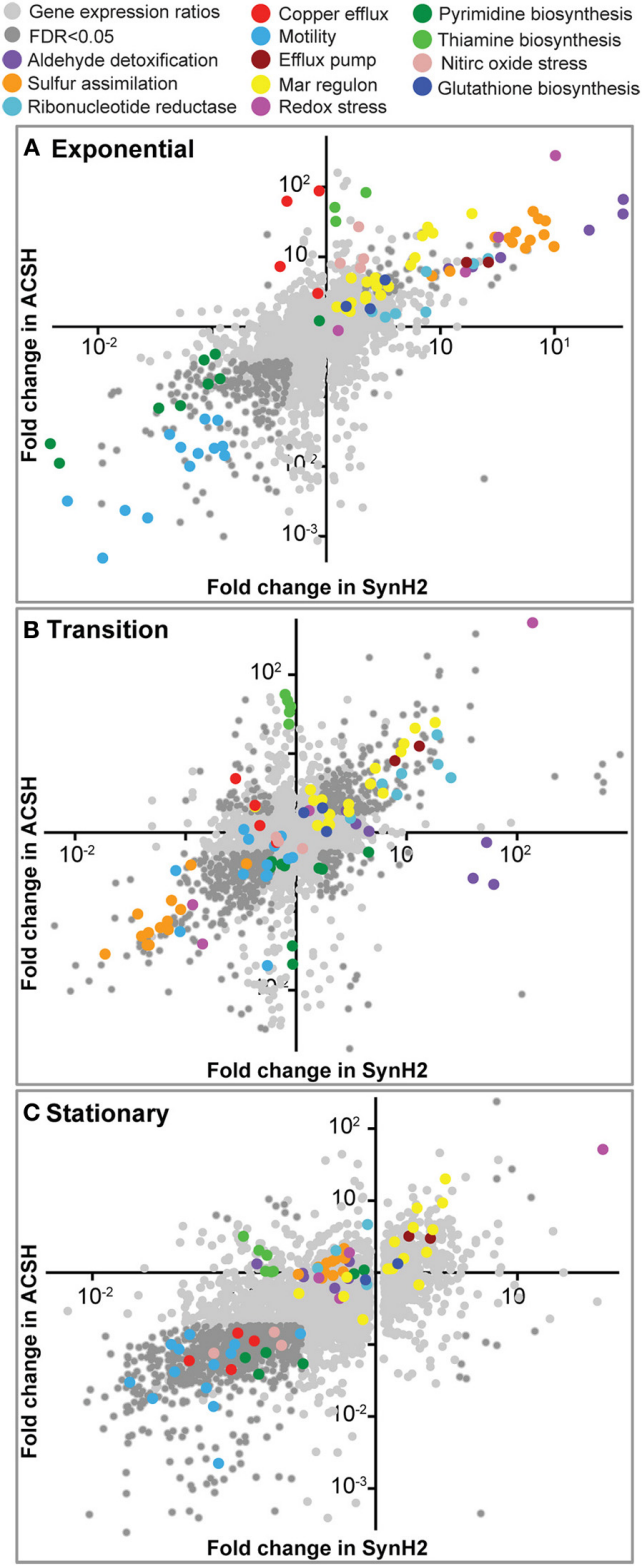
**Relative gene expression patterns in SynH2 and ACSH cells relative to SynH2^−^ cells**. Scatter plots were prepared with the ACSH/SynH2^−^ gene expression ratios plotted on the y-axis and the SynH2/SynH2^−^ ratios on the x-axis (both on a log_10_ scale). GLBRCE1 was cultured in a bioreactor anaerobically (Figure 1 and Figure S5); RNAs were prepared from exponential **(A)**, transition **(B)**, or stationary **(C)** phase cells and subjected to RNA-seq analysis (Materials and Methods). Dark gray dots represent genes for which *p* = 0.05 for each expression ratio. Sets of genes with related functions that exhibited significant discrepant or parallel changes are color-coded and described in the legend at the top (see also Tables S3, S4, respectively).

To test the similarity of SynH2 to ACSH and the extent to which LC-derived inhibitors impact ethanologenesis, we next used RNA-seq to compare gene expression patterns of GLBRCE1 grown in the two media relative to cells grown in SynH2^−^ (Materials and Methods; Table 1). We computed normalized gene expression ratios of ACSH cells vs. SynH2^−^ cells and SynH2 cells vs. SynH2^−^ cells, and then plotted these ratios against each other using log_10_ scales for exponential phase (Figure 2A), transition phase (Figure 2B), and stationary phase (Figure 2C). For simplicity, we refer to these comparisons as the SynH2 and ACSH ratios. The SynH2 and ACSH ratios were highly correlated in all three phases of growth, although were lower in transition and stationary phases (Pearson's *r* of 0.84, 0.66, and 0.44 in exponential, transition, and stationary, respectively, for genes whose SynH2 and ACSH expression ratios both had corrected *p* < 0.05; *n* = 390, 832, and 1030, respectively). Thus, SynH2 is a reasonable mimic of ACSH.

We used these data to investigate the gene expression differences between SynH2 and ACSH (Table S3). Several differences likely reflected the absence of some trace carbon sources in SynH2 (e.g., sorbitol, mannitol), their presence in SynH2 at higher concentrations than found in ACSH (e.g., citrate and malate), and the intentional substitution of D-arabinose for L-arabinose. Elevated expression of genes for biosynthesis or transport of some amino acids and cofactors confirmed or suggested that SynH2 contained somewhat higher levels of Trp, Asn, thiamine and possibly lower levels of biotin and Cu^2+^ (Table S3). Although these discrepancies point to minor or intentional differences that can be used to refine the SynH recipe further, overall we conclude that SynH2 can be used to investigate physiology, regulation, and biofuel synthesis in microbes in a chemically defined, and thus reproducible, media to accurately predict behaviors of cells in real hydrolysates like ACSH that are derived from ammonia-pretreated biomass.

### Aromatic aldehydes in SynH2 are converted to alcohols, but phenolic carboxylates and amides are not metabolized

**Figure 3:**
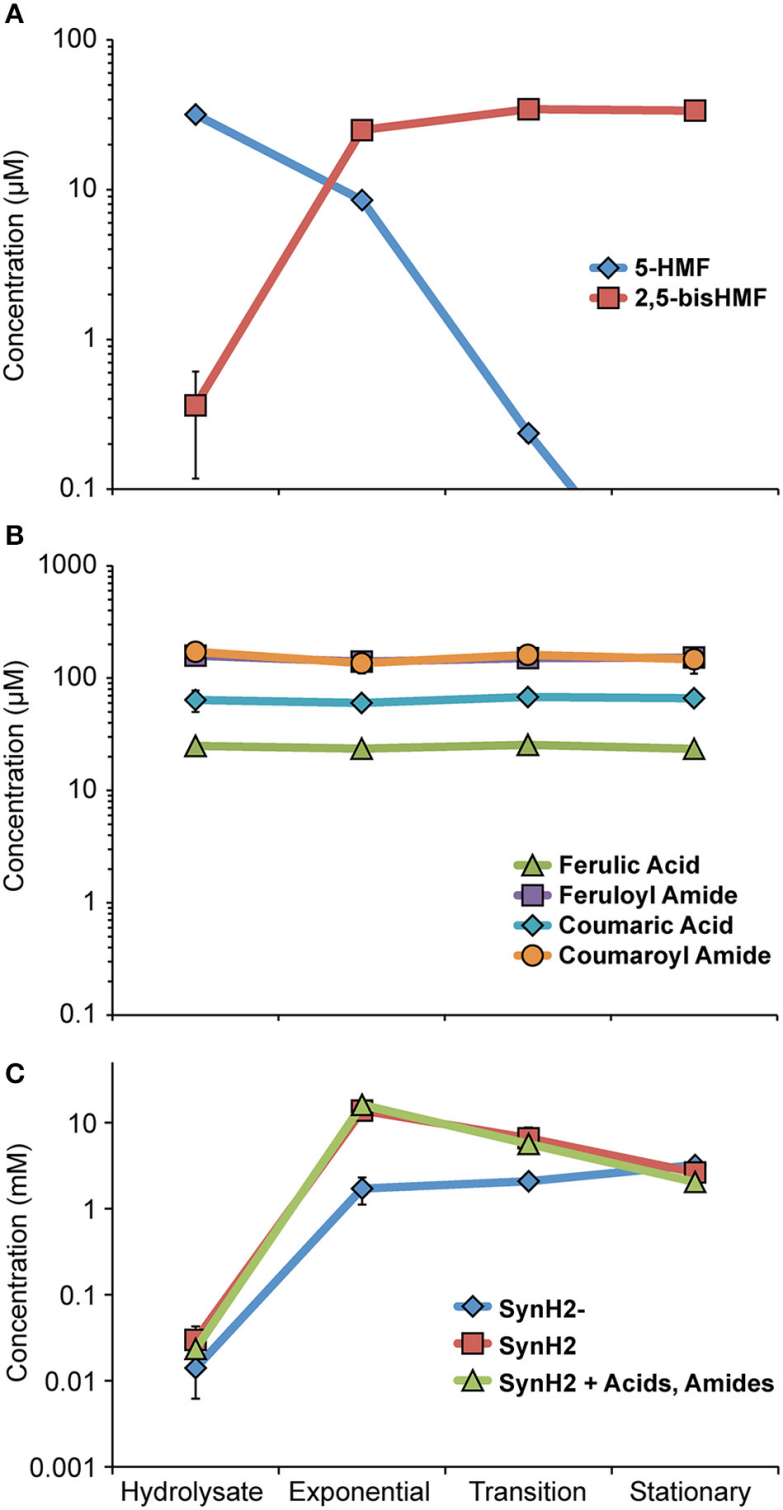
**Growth phase-dependent changes in SynH2 aromatic inhibitor levels**. GLBRCE1 was cultured under anaerobic conditions in SynH2 in bioreactors. Levels of the major LC-derived inhibitors in the culture medium were determined as described in Materials and Methods. “Hydrolysate” refers to medium immediately prior to inoculation, “Exp,” “Trans,” and “Stat” refers to samples collected during exponential, transition, and stationary phase growth, respectively. **(A)** Metabolic fate of hydroxymethylfurfural (HMF). Concentrations of HMF and 2,5-bis-HMF (2,5-bis-hydroxymethylfurfuryl alcohol) are represented. **(B)** Metabolic fates of the major aromatic acids and amides. Concentrations of ferulic acid, feruloyl amide, coumaric acid, and coumaroyl amide are shown. **(C)** Concentration of acetaldehyde in the culture medium when GLBRCE1 was grown in SynH2, SynH2^−^, or SynH2 with aromatic aldehydes only omitted.

Before evaluating how patterns of gene expression informed the physiology of GLBRCE1 in SynH2, we first determined the profiles of inhibitors, end-products, and intracellular metabolites during ethanologenesis. The most abundant aldehyde inhibitor, HMF, quickly disappeared below the limit of detection as the cells entered transition phase with concomitant and approximately stoichiometric appearance of the product of HMF reduction, 2,5-bis-HMF (hydroxymethylfurfuryl alcohol; Figure 3A, Table S8). Hydroxymethylfuroic acid did not appear during the fermentation, suggesting that HMF is principally reduced by aldehyde reductases such as YqhD and DkgA, as previously reported for HMF and furfural generated from acid-pretreated biomass (Miller et al., 2009a, 2010; Wang et al., 2013). In contrast, the concentrations of ferulic acid, coumaric acid, feruloyl amide, and coumaroyl amide did not change appreciably over the course of the experiment (Figure 3B, Table S8), suggesting that *E. coli* either does not encode activities for detoxification of phenolic carboxylates and amides, or that expression of such activities is not induced in SynH2.

Although HMF disappeared early in fermentation, acetaldehyde accumulated to >10 mM during exponential and transition phase in both SynH2 and ACSH (Figure 3C, Table S8). Elevated acetaldehyde relative to SynH2^−^ was also observed upon omission of aromatic aldehydes from SynH2, demonstrating that LC-derived phenolic acids and amides alone can cause accumulation of acetaldehyde (Figure 3C). Thus, acetaldehyde accumulation was not simply a consequence of diverting reducing equivalents to detoxification of the aromatic aldehydes like HMF but likely resulted from a broader impact of LC-derived inhibitors on cellular energetics that decreased the pools of NADH available for conversion of acetaldehyde to ethanol.

### Lignocellulose-derived inhibitors negatively impact carbon and energy metabolism, resulting in accumulation of pyruvate and acetaldehyde

**Figure 4:**
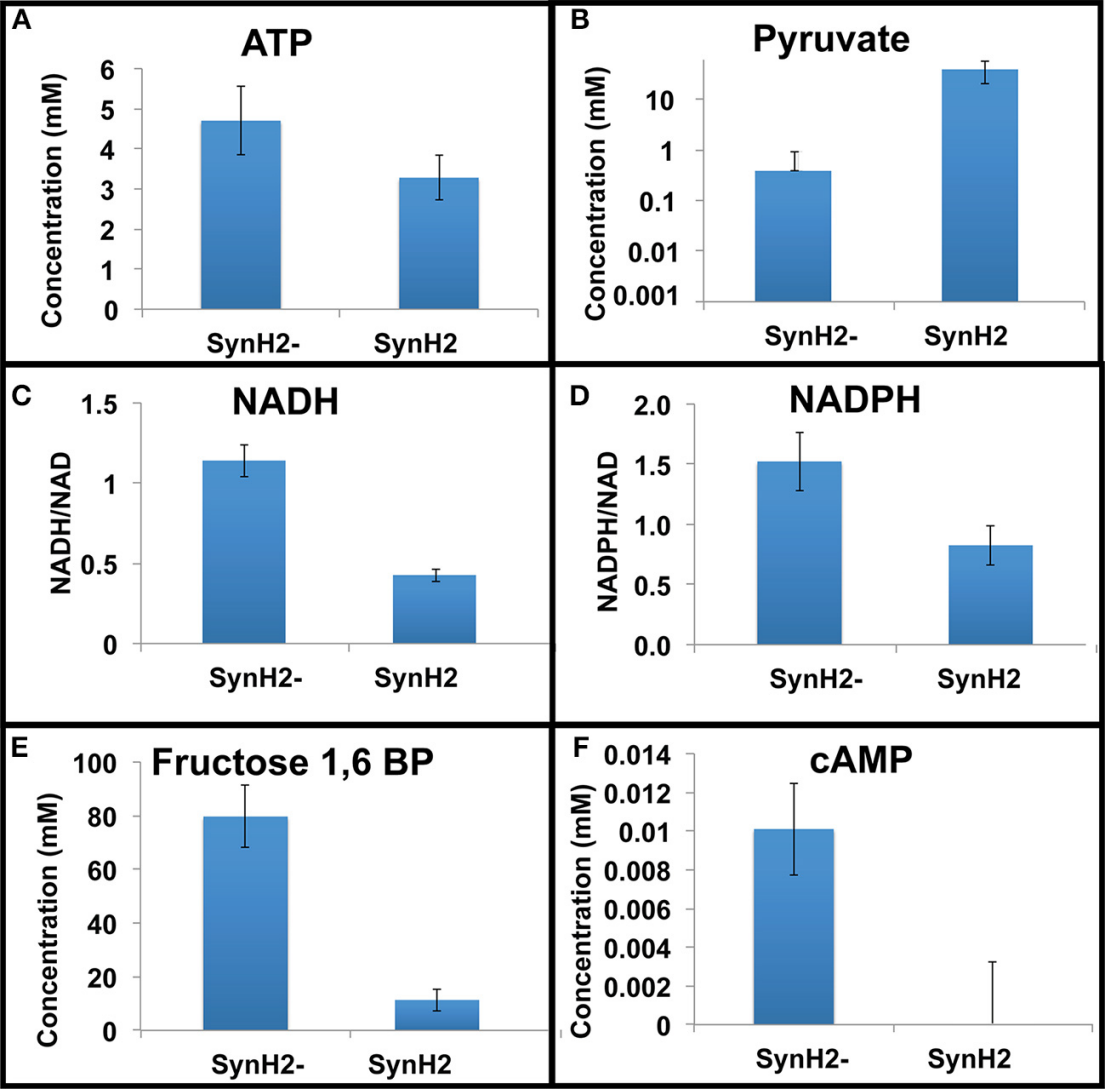
**Relative metabolite levels in SynH2 and SynH2^−^ cells**. GLBRCE1 was cultured anaerobically in bioreactors in SynH2 and SynH2^−^. Metabolites were prepared from exponential phase cells and analyzed as described in the Material and Methods. Shown are intracellular concentrations of ATP **(A)**, pyruvate **(B)**, fructose-1,6-bisphosphate **(E)**, and cAMP (**F**). **(C,D)** show the ratios of NADH/NAD^+^ and NADPH/NADP^+^, respectively.

Examination of intracellular metabolites revealed that aromatic inhibitors decreased the levels of metabolites associated with glycolysis and the TCA cycle (Figures 4B,E; Table S1). Strikingly, metabolites associated with cellular energetics and redox state were also decreased in SynH2 cells relative to SynH2^−^ cells (Figures 4A,C,D,F; Table S1). ATP was reduced 30%; the NADH/NAD^+^ ratio decreased by 63%; and the NADPH/NADP^+^ ratio decreased 56%. Together, these data indicate that the aromatic inhibitors dramatically decreased cellular energy pools and available reducing equivalents in SynH2 cells. The consequences of energetic depletion were readily apparent with an approximate 100-fold increase in the intracellular levels of pyruvate in SynH2 cells (to ~14 mM), despite the disappearance of pyruvate from the growth medium (Table S1, Figure 4B, and data not shown). The increase in pyruvate and correspondingly in acetaldehyde (Figures 3C, 4B) suggest that the reduced rate of glucose-to-ethanol conversion caused by aromatic inhibitors results from inadequate supplies of NADH to convert acetaldehyde to ethanol.

Transition-phase SynH2 vs. SynH2^−^ cells exhibited similar trends in aromatic-inhibitor-dependent depletion of some glycolytic intermediates, some TCA intermediates, and ATP, along with elevation of pyruvate and acetaldehyde (Table S1; Figure 3C). Stationary phase cells displayed several differences, however. Glycolytic intermediates (glucose 6-phosphate, fructose 6-phosphate, fructose 1,6 diphosphate, and 2-, 3-phosphoglycerate) were approximately equivalent in SynH2 and SynH2^−^ cells, whereas pyruvate concentrations dropped significantly (Table S1). The impact of the inhibitors was largely attributable to the phenolic carboxylate and amides alone, as removal of the aldehydes from SynH2 changed neither the depletion of glycolytic and TCA intermediates nor the elevation of pyruvate and acetaldehyde (data not shown). We conclude that phenolic carboxylates and amides in SynH2 and ACSH have major negative impacts on the rate at which cells grow and consequently can convert glucose to ethanol.

### Aromatic inhibitors induce gene expression changes reflecting energy stress

Given the major impacts of aromatic inhibitors on ethanologenesis, we next sought to address how these inhibitors impacted gene expression and regulation in *E. coli* growing in SynH2. To that end, we first identified pathways, transporters, and regulons with similar relative expression patterns in SynH2 and ACSH using both conventional gene set enrichment analysis and custom comparisons of aggregated gene expression ratios (Materials and Methods). These comparisons yielded a curated set of regulons, pathways, and transporters whose expression changed significantly in SynH2 or ACSH relative to SynH2^−^ (aggregate *p* < 0.05; Table S4).

For many key pathways, transporters, and regulons, similar trends were seen in both SynH2 and ACSH vs. SynH2^−^ (Figure 2 and Table S4). The most upregulated gene sets reflected key impacts of aromatic inhibitors on cellular energetics. Anabolic processes requiring a high NADPH/NADP^+^ potential were significantly upregulated (e.g., sulfur assimilation and cysteine biosynthesis, glutathione biosynthesis, and ribonucleotide reduction). Additionally, genes encoding efflux of drugs and aromatic carboxylates (e.g., *aaeA*) and regulons encoding efflux functions (e.g., the *rob* regulon), were elevated. Curiously, both transport and metabolism of xylose were downregulated in all three growth phases in both media, suggesting that even prior to glucose depletion aromatic inhibitors reduce expression of xylose genes and thus the potential for xylose conversion. Currently the mechanism of this repression is unclear, but it presumably reflects either an indirect impact of altered energy metabolism or an interaction of one or more of the aromatic inhibitors with a regulator that decreases xylose gene expression.

During transition phase, a different set of genes involved in nitrogen assimilation were upregulated in SynH2 cells and ACSH cells relative to SynH2^−^ cells (Table S5). Previously, we found that transition phase corresponded to depletion of amino acid nitrogen sources (e.g., Glu and Gln; Schwalbach et al., 2012). Thus, this pattern of aromatic-inhibitor-induced increase in the expression of nitrogen assimilation genes during transition phase suggests that the reduced energy supply caused by the inhibitors increased difficulty of ATP-dependent assimilation of ammonia. Interestingly, the impact on gene expression appeared to occur earlier in ACSH than in SynH2, which may suggest that availability of organic nitrogen is even more growth limiting in ACSH.

**Figure 5:**
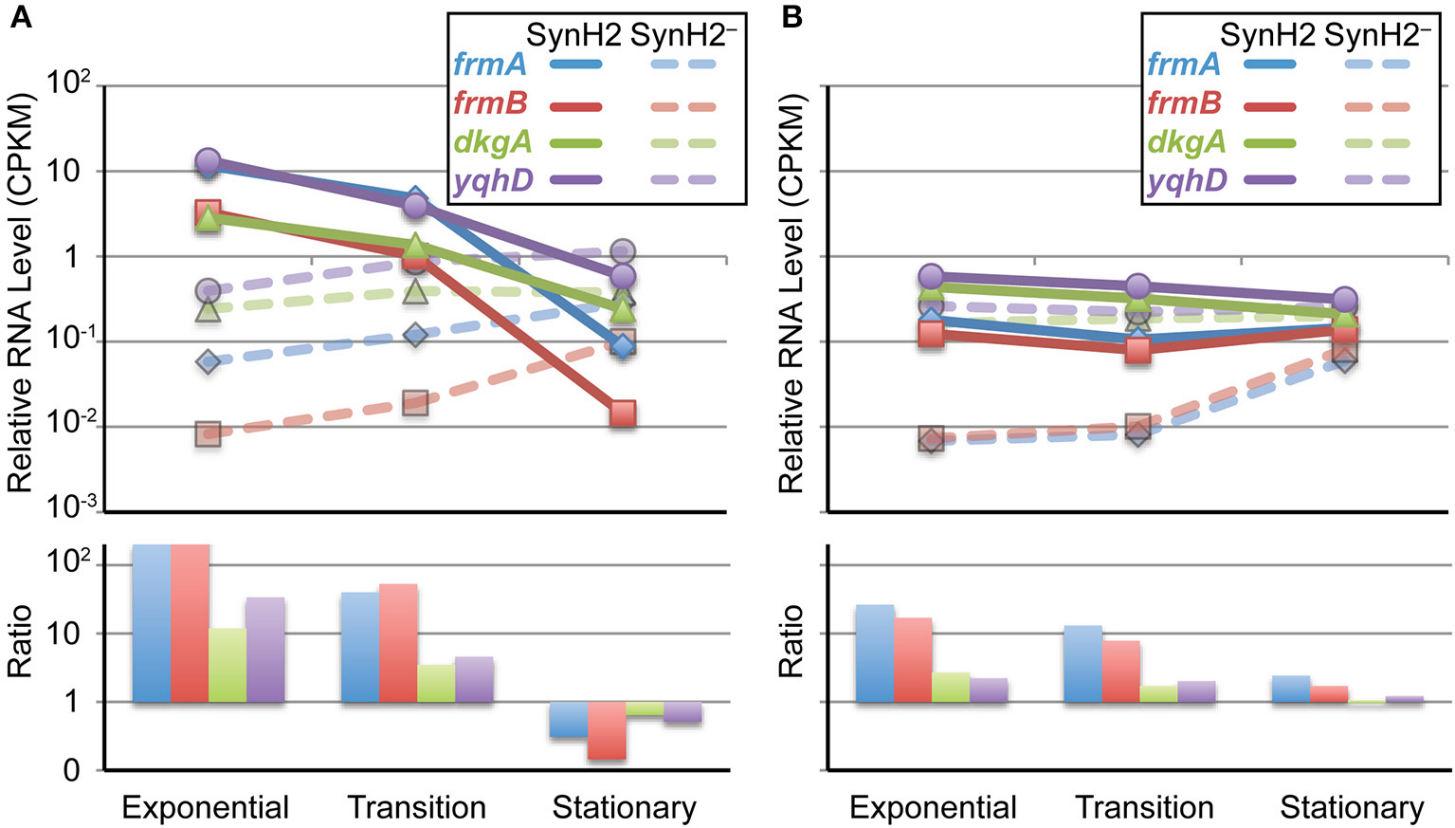
**Growth phase-dependent changes in inhibitor-responsive gene expression**. Changes in RNA levels for genes that comprise the major regulatory response to aromatic inhibitors in SynH2. Shown are normalized RNA-seq measurements (top panel) from GLBRCE1 grown in SynH2 (solid lines) or SynH2^−^ (dotted lines) or their relative ratios (bottom panel) from exponential, transition, and stationary phases of growth as indicated. **(A)** Aldehyde detoxification genes (*frmA, frmB, dkgA*, and *yqhC*). **(B)** Genes that encode efflux pumps (*aaeA, aaeB, acrA, acrB*).

Of particular interest were the patterns of changes in gene expression related to the detoxification pathways for the aromatic inhibitors. Our gene expression analysis revealed inhibitor induction of genes encoding aldehyde detoxification pathways (*frmA*, *frmB*, *dkgA*, and *yqhD*) that presumably target LC-derived aromatic aldehydes (e.g., HMF and vanillin) and acetaldehyde that accumulates when NADH-dependent reduction to ethanol becomes inefficient (Herring and Blattner, 2004; Gonzalez et al., 2006; Miller et al., 2009b, 2010; Wang et al., 2013) as well as efflux pumps controlled by MarA/SoxS/Rob (e.g., *acrA* and *acrB*) and the separate system for aromatic carboxylates (*aaeA* and *aaeB*) (Van Dyk et al., 2004). Interestingly, we observed that expression of the aldehyde detoxification genes *frmA*, *frmB*, *dkgA*, and *yqhD* paralleled the levels of LC-derived aromatic aldehydes and acetaldehyde detected in the media (Figure 3). Initially high-level expression was observed in SynH2 cells, which decreased as the aldehydes were inactivated (Figure 5A). Conversely, expression of these genes increased in SynH2^−^ cells, surpassing the levels in SynH2 cells in stationary phase when the level of acetaldehyde in the SynH2^−^ culture spiked past that in the SynH2 culture. The elevation of *frmA* and *frmB* is particularly noteworthy as the only reported substrate for FrmAB is formaldehyde. We speculate that this system, which has not been extensively studied in *E. coli*, may also act on acetaldehyde. Alternatively, formaldehyde, which we did not assay, may have accumulated in parallel to acetaldehyde.

In contrast to the decrease in *frmA*, *frmB*, *dkgA*, and *yqhD* expression as SynH2 cells entered stationary phase, expression of *aaeA*, *aaeB*, *acrA*, and *acrB* remained high (Figure 5B). This continued high-level expression is consistent with the persistence of phenolic carboxylates and amides in the SynH2 culture (Figure 3), and presumably reflect the futile cycle of antiporter excretion of these inhibitors to compete with constant leakage back into cells.

### Post-transcriptional effects of aromatic inhibitors were limited primarily to stationary phase

**Figure 6:**
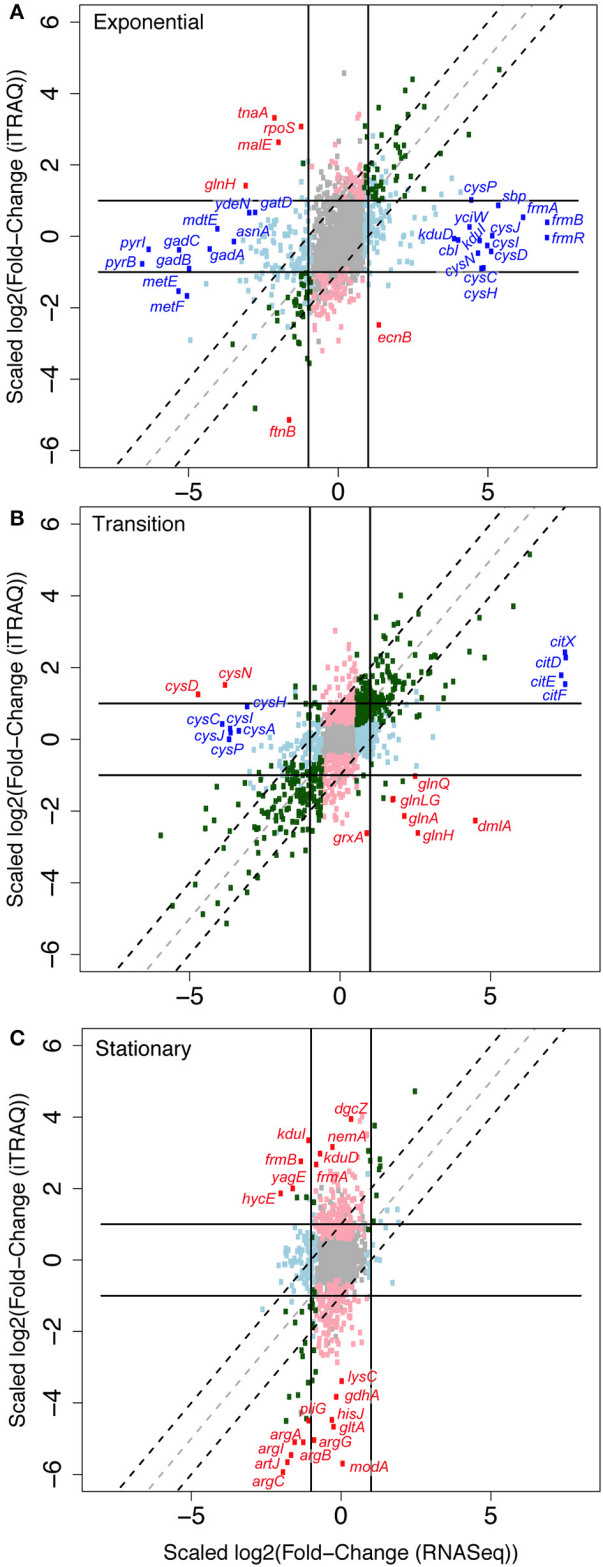
**Effects of aromatic inhibitors on protein levels compared to effects on cognate RNA levels**. Scatter plot comparing log_2_-fold RNA ratios (x-axis) to log_2_-fold protein ratios (y-axis) of GLBRCE1 genes and gene products for cells for grown in SynH2 compared to the reference medium, SynH2^−^. Cells were collected and proteomic samples prepared from exponential **(A)**, transition **(B)**, and stationary **(C)** growth phases. The lines indicate boundaries beyond which changes exceed 2-fold. The dotted lines demarcate the area expected for parallel changes in protein and RNA levels. *Red*, genes for which changes in protein levels were not paralleled by changes in the corresponding RNA and for which the discrepancy had a *p* ≤ 0.05 (see Table S7). *Blue*, genes for which changes in RNA levels were not paralleled by changes in the corresponding protein and for which the discrepancy had a *p* ≤ 0.05. *Gray*, *p* > 0.05 for both RNA and protein ratios. *Light blue*, *p* ≤ 0.05 for RNA ratio but not for protein ratio. *Light* pink, *p* ≤ 0.05 for protein ratio but not for RNA ratio. *Green*, *p* ≤ 0.05 for both RNA and protein ratios and effects are parallel.

We next investigated the extent to which the aromatic inhibitors could exert effects on cellular regulation post-transcriptionally rather than *via* transcriptional regulators by comparing inhibitor-induced changes in protein levels to changes in RNA levels. For this purpose, we used iTRAQ quantitative proteomics to assess changes in protein levels (Material and Methods). We then normalized the log_2_-fold-changes in protein levels in each of the three growth phases to changes in RNA levels determined by RNA-seq and plotted the normalized values against each other (Figures 6A–C; Tables S6, S7). Most proteome and transcriptome fold-changes fall within a factor of 2 of the diagonal, consistent with concordant changes in mRNA and protein and thus limited post-transcriptional effects of aromatic inhibitors. A small number of RNA-protein pairs exhibited an >2-fold change with *p* < 0.05. During exponential phase, four proteins were present at elevated levels relative to changes in RNA levels, which actually decreased (RpoS, TnaA, MalE, and GlnH; red circles, Figure 6A; Table S7A), whereas 26 RNAs increased or decreased significantly with little difference in proteins levels (blue circles, Figure 6A; Table S7A). These disparate increases in RNA levels included some of the major transcriptional responses to the inhibitors (S assimilation and the FrmA aldehyde detoxification pathway), and these proteins were present at high levels both with and without inhibitors (Table S7D). Several observations led us to conclude that these discrepancies in protein and RNA levels between SynH2^−^ and SynH2 cells reflect induction of expression in SynH2 cells but carryover of elevated protein levels in the inoculum of SynH2^−^ cells not yet diluted in exponential phase. First, we sampled exponential phase between one and two cell doublings so that proteins elevated in stationary phase in the inoculum might still be present. Second, FrmRAB and S assimilation genes are elevated in stationary SynH2^−^ cells relative to SynH2 cells (Table S7C), likely reflecting the greater accumulation of acetaldehyde in SynH2^−^ cells in stationary phase (Figure 3C). Finally, RpoS and TnaA are markers of stationary phase (Lacour and Landini, 2004) and may reflect elevation of these proteins in SynH stationary cells carried over from the inoculum. In a similar vein, the apparent overrepresentation of PyrBI, GadABC, and MetEF proteins in SynH2 cells could reflect their greater abundance in stationary phase SynH2 cells that were carried over to early exponential phase.

Supporting this view, transition phase cells in which the inoculum was diluted >5-fold exhibited a higher correlation between protein and RNA levels and only limited evidence of post-transcriptional regulation caused by the aromatic inhibitors (Figure 6B). Three clusters of outliers reflected (i) reduced transcript levels for S assimilation genes in SynH2^−^ without a corresponding drop in protein level (*cys* genes), (ii) higher levels of *glnAGHLQ* transcripts in SynH2 cells than SynH2^−^ cells with high protein levels in both, and (iii) high induction of transcripts for the citrate assimilation system (*citDEFX*) in SynH2 with lesser induction of protein levels. These effects likely reflect adjustment of S assimilation gene expression during transition phase, a greater induction of N assimilation in the more rapidly growing SynH2 cells, and induction of citrate assimilation by the aromatic inhibitors.

The clearest evidence for post-transcriptional regulation caused by the aromatic inhibitors appeared in stationary phase (Figure 6C). A set of proteins involved in arginine, glutamate, lysine and citrate biosynthesis (ArgABCGI, GdhA, LysC, GltA) and periplasmic proteins arginine high-affinity import (ArtJ), histidine high-affinity import (HisJ), molybdate import (ModA), and lysozyme inhibition (PliG) decreased dramatically in SynH2 cells relative to SynH2^−^ cells without corresponding reductions of their transcripts. GdhA, other biosynthetic enzymes, and other periplasmic binding proteins are degraded by the ClpP protease during C or N starvation (Maurizi and Rasulova, 2002; Weichart et al., 2003); Lon protease also has been implicated in proteolysis upon C starvation (Luo et al., 2008). Thus, we suggest that aromatic inhibitors may enhance degradation of proteins involved in N and C metabolism in stationary phase cells. The periplasmic proteins must be degraded as precursors or mediated by an additional effect involving periplasmic proteases.

## Discussion

**Figure 7:**
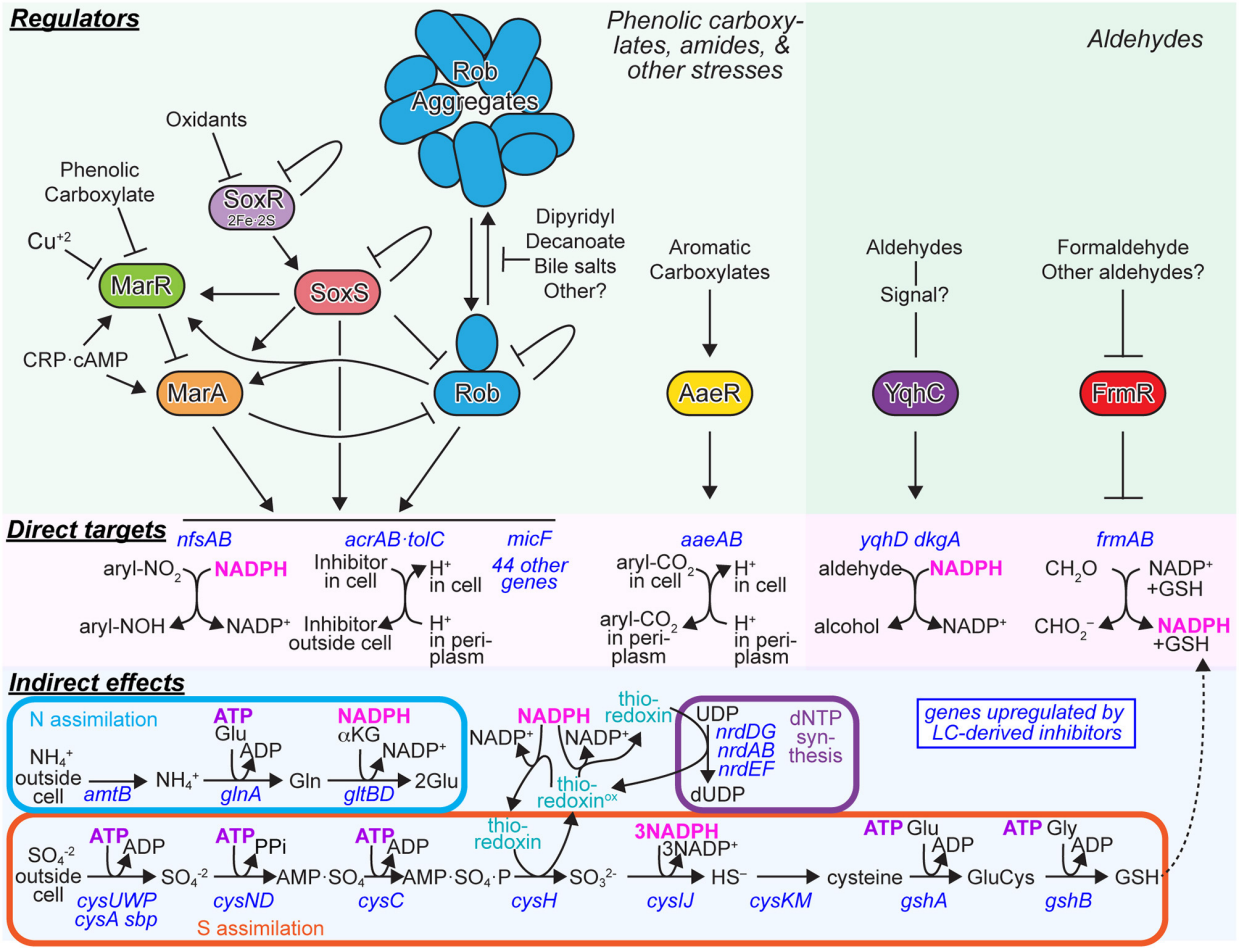
**Major Regulatory responses of *E. coli* to aromatic inhibitors found in ACSH**. The major *E. coli* responses to phenolic carboxylates and amides (left) or responses to aldehydes (right) are depicted. *Green panels*, regulators and signaling interactions that mediate the regulatory responses. *Pink panels*, direct targets of the regulators that consume reductant (NADPH) for detoxification reactions or deplete the proton motive force through continuous antiporter efflux of aromatic carboxylates. *Blue panels*, indirect effects of inhibitors mediated by reductions in ATP and NADPH levels.

Results of our investigation into the effects of LC-derived inhibitors on *E. coli* ethanologenesis support several key conclusions that will guide future work. First, a chemically defined mimic of ACSH (SynH2) that contained the major inhibitors found by chemical analysis of ACSH adequately replicated both growth and the rates of glucose and xylose conversion to ethanol by *E. coli*. SynH2-replication of ACSH required inclusion of osmolytes found in ACSH and established that, at the ratios present in ACSH, phenolic carboxylates and amides, which are not metabolized by *E. coli*, had a greater overall impact on cell growth than phenolic aldehydes and furfurals, which were metabolized. In both SynH2 and ACSH, *E. coli* entered a metabolically active stationary phase as cells exhausted organic sources of N and S (e.g., amino acids) and during which the inhibitors greatly reduced xylose conversion. The impact of inhibitors on cellular energetics reduced levels of ATP, NADH, and NADPH and was seen most dramatically for energetically challenging processes requiring NADPH (like SO^−2^_4_ assimilation and deoxyribonucleotide production), during transition to the stationary phase on ATP-dependent NH_3_ assimilation, and in elevated pyruvate levels presumably reflecting reduced NADH-dependent flux of pyruvate to ethanol (Figure 7). The direct effects of the inhibitors on cells appear to be principally mediated by transcriptional rather than translational regulators, with the MarA/SoxS/Rob network, AaeR, FrmR, and YqhC being the most prominent players. Although the effect of the inhibitors on transcriptional regulation of the efflux pumps was striking, increased efflux activity itself may perturb cellular metabolism. For example, Dhamdhere and Zgurskaya (2010) have shown that deletion of the AcrAB-TolC complex results in metabolic shutdown and high NADH/NAD^+^ ratios. By analogy, overexpression of efflux pumps may have the opposite effect (e.g., lowering of NADH/NAD^+^ ratios), which is consistent with observations in this study. In addition, recent work suggests that the *acrAB* promoter is upregulated in response to certain cellular metabolites (including those related to cysteine and purine biosynthesis), which are normally effluxed by this pump (Ruiz and Levy, 2014). Therefore, upregulation of AcrAB-TolC may impact homeostatic mechanisms of cellular biosynthetic pathways, resulting in continuous upregulation of pathways that require large amounts of reducing power in the form of NADPH. It is also possible that LC-derived inhibitors perturb metabolism directly in ways that generate additional AcrAB-TolC substrates, potentially increasing energy-consuming efflux further. Given these intricacies, further studies to unravel the mechanistic details of the effects of efflux pump activity on cellular metabolism, as a result of exposure to LC-derived inhibitors, are warranted.

The inability of cells to convert xylose in the presence of inhibitors appears to result from a combination of both effects on gene expression and some additional effect on transport or metabolism. The inhibitors lowered xylose gene expression (XylR regulon; *xylABFGH*) by a factor of 3-5 during all three growth phases (Table S4). This effect was not caused by the previously documented AraC repression (Desai and Rao, 2010), since it persisted in SynH2 when we replaced the AraC effector L-arabinose with D-arabinose, but might reflect lower levels of cAMP caused by the inhibitors (Figure 4); both the *xylAB* and *xylFGH* operons are also regulated by CRP·cAMP. Nonetheless, significant levels of XylA, B, and F were detected even in the presence of inhibitors (Table S7D), even though xylose conversion remained inhibited even after glucose depletion (Table 2). Thus, the inability to convert xylose may also reflect either the overall impact of inhibitors on cellular energetics somehow making xylose conversion unfavorable or an effect of xylose transport or metabolism that remains to be discovered. Further studies of the impact of inhibitors on xylose transport and metabolism are warranted. It would be particularly interesting to test SynH formulations designed to compare the conversion efficiencies of xylose, arabinose, and C6 sugars other than glucose.

The central focus of this study was to understand the impact of inhibitors of gene expression regulatory networks. The apparent lack of involvement of post-transcriptional regulation suggests that *E. coli* mounts a defense against LC-derived inhibitors principally by controlling gene transcription, probably reflecting evolution of specific bacterial responses to LC-derived inhibitors. Although enteric bacteria do not ordinarily encounter industrial lignocellulosic hydrolysates, they likely encounter the same suite of compounds from digested plant material in the mammalian gut. Thus, evolution of specific responses is reasonable. A key question for future studies is whether phenolic amides, not ordinarily present in digested biomass, will also invoke these responses in the absence of carboxylates or aldehydes. We note that the apparent absence of a translational regulatory response in the cellular defense against LC-derived inhibitors does not preclude involvement of either direct or indirect post-transcriptional regulation in fine-tuning the response. Our proteomic measurements would likely not have detected fine-tuning. Additionally, we did detect an apparently indirect induction by inhibitors of protein degradation in stationary phase, possibly in response to C starvation (Figure 6C). Finally, we note that the sRNA micF, a known post-transcriptional regulator, is a constituent of the MarA/SoxS/Rob regulon and was upregulated by inhibitors. Although confidence was insignificant due to poor detection of sRNAs in RNAseq data, the induction of micF was confirmed in a separate study of sRNAs (Ong and Landick, in preparation). Thus, a more focused study of the involvement of sRNAs in responses to LC inhibitors would likely be informative.

MarA/SoxS/Rob is a complex regulon consisting of the three inter-connected primary AraC-class regulators that bind as monomers to 20-bp sites in promoters with highly overlapping specificity and synergistically regulate ~50 genes implicated in resistance to multiple antibiotics and xenobiotics, solvent tolerance, outer membrane permeability, DNA repair, and other functions (Chubiz et al., 2012; Duval and Lister, 2013; Garcia-Bernardo and Dunlop, 2013) (Figure 7). Twenty-three genes, including those encoding the AcrAB·TolC efflux pump, the NfsAB nitroreductases, the micF sRNA, superoxide dismutase, some metabolic enzymes (e.g., Zwf, AcnA, and FumC) and incompletely characterized stress proteins are controlled by all three regulators, whereas other genes are annotated as being controlled by only a subset of the regulators (Duval and Lister, 2013), www.ecocyc.org; (Keseler et al., 2013). MarA and SoxS lack the C-terminal dimerization domain of AraC; this domain is present on Rob and appears to mediate regulation by aggregation that can be reversed by effectors (Griffith et al., 2009). Inputs capable of inducing these genes, either through the MarR and SoxR repressors that control MarA and SoxS, respectively, or by direct effects on Rob include phenolic carboxylates, Cu^2+^, a variety of organic oxidants, dipyridyl, decanoate, bile salts, Fis, and Crp·cAMP (Martin and Rosner, 1997; Rosner et al., 2002; Rosenberg et al., 2003; Chubiz and Rao, 2010; Duval and Lister, 2013; Hao et al., 2014) (Figure 7). Given these diverse inputs, it seems highly likely that ferulate and coumarate in ACSH induce the MarA/SoxS/Rob regulon via MarR. Indeed, LC-hydrolysate and ferulate induction of MarA has been reported (Lee et al., 2012). Interestingly, Cu^2+^ recently was shown to induce MarR by oxidation to create MarR disulfide dimer (Hao et al., 2014). Given the elevated levels of Cu^2+^ in ACSH reflected by induction of Cu^2+^ efflux (Figure 2; Table S4), induction of MarA/SoxS/Rob in ACSH may result from synergistic effects of Cu^2+^ and phenolic carboxylates, oxidants that affect SoxR, and yet-to-be-determined compounds that affect Rob. A second response in LC-derived inhibitors appears to be mounted by the LysR-type regulator AaeR, which controls the AaeAB aromatic carboxylate efflux system (Van Dyk et al., 2004) (Figure 7). Both phenolic and aryl carboxylates induce AaeAB through AaeR, but little is known about its substrate specificity or mechanism of activation.

Two distinct regulators, YqhC and FrmR, control synthesis of the YqhD/DkgA NAPDH-dependent aldehyde reductases and the FrmAB formaldehyde oxidase, respectively (Herring and Blattner, 2004; Turner et al., 2011). Even less is known about these regulators, although the DNA-binding properties of YqhC have been determined. In particular, it is unclear how aldehydes cause induction, although the current evidence suggests effects on YqhC are likely to be indirect. Given the central role of the regulators AaeR, YqhC, and FrmR in the cellular response to LC-derived inhibitors, further study of their properties and mechanisms is likely to be profitable. With sufficient understanding and engineering, they could be used as response regulators to engineer cells that respond to LC-inhibitors in ways that maximize microbial conversion of sugars to biofuels.

What types of responses would optimize biofuel synthesis? It appears the naturally evolved responses, namely induction of efflux systems and NADPH-dependent detoxification pathways, may not be optimal for efficient synthesis of biofuels. We infer this conclusion for several reasons. First, our gene expression results reveal that crucial pathways for cellular biosynthesis that are among the most energetically challenging processes in cells, S assimilation, N assimilation, and ribonucleotide reduction, are highly induced by LC-derived inhibitors (Figures 2, 7; Table S4). A reasonable conjecture is that the diversion of energy pools, including NADPH and ATP, to detoxification makes S assimilation, N assimilation, and ribonucleotide reduction difficult, increasing expression of genes for these pathways indirectly. The continued presence of the phenolic carboxylates and amides (Figure 3) likely causes futile cycles of efflux. As both the AcrAB and AaeAB efflux pumps function as proton antiporters (Figure 7), continuous efflux is expected to decrease ATP synthesis by depleting the proton-motive force. Although this response makes sense evolutionarily because it protects DNA from damage by xenobiotics, it does not necessarily aid conversion of sugars to biofuels. Disabling these efflux and detoxification systems, especially during stationary phase when cell growth is no longer necessary, could improve rates of ethanologenesis. Indeed, Ingram and colleagues have shown that disabling the NADPH-dependent YqhD/DkgA enzymes or better yet replacing them with NADH-dependent aldehyde reductases (e.g., FucO) can improve ethanologenesis in furfural-containing hydrolysates of acid-pretreated biomass (Wang et al., 2011a, 2013). That simply deleting *yqhD* improves ethanologenesis argues that, in at least some cases, it is better to expose cells to LC-derived inhibitors than to spend energy detoxifying the inhibitors.

Some previous efforts to engineer cells for improved biofuel synthesis have focused on overexpression of selected efflux pumps to reduce the toxic effects of biofuel products (Dunlop et al., 2011). Although this strategy may help cells cope with the effects of biofuel products, our results suggest an added potential issue when dealing with real hydrolysates, namely that efflux pumps may also reduce the rates of biofuel yields by futile cycling of LC-derived inhibitors. Thus, effective use of efflux pumps will require careful control of their synthesis (Harrison and Dunlop, 2012). An alternative strategy to cope with LC-derived inhibitors may be to devise metabolic routes to assimilate them into cellular metabolism.

In conclusion, our findings illustrate the utility of using chemically defined mimics of biomass hydrolysates for genome-scale study of microbial biofuel synthesis as a strategy to identify barriers to biofuel synthesis. By identifying the main inhibitors present in ammonia-pretreated biomass hydrolysate and using these inhibitors in a synthetic hydrolysate, we were able to identify the key regulators responsible for the cellular responses that reduced the rate of ethanol production and limited xylose conversion to ethanol. Knowledge of these regulators will enable design of new control circuits to improve microbial biofuel production.

### Conflict of interest statement

The authors declare that the research was conducted in the absence of any commercial or financial relationships that could be construed as a potential conflict of interest.
