# Supplementary material for: Aromatic inhibitors derived from ammonia-pretreated lignocellulose hinder bacterial ethanologenesis by activating regulatory circuits controlling inhibitor efflux and detoxification
Source: Front Microbiol. 2014 Aug 13;5:402. doi: 10.3389/fmicb.2014.00402 (PMC4132294; doi:10.3389/fmicb.2014.00402)
Supplement: Supplementary file 1 [file DataSheet1.ZIP › Figure S1.pdf]

Figure S1

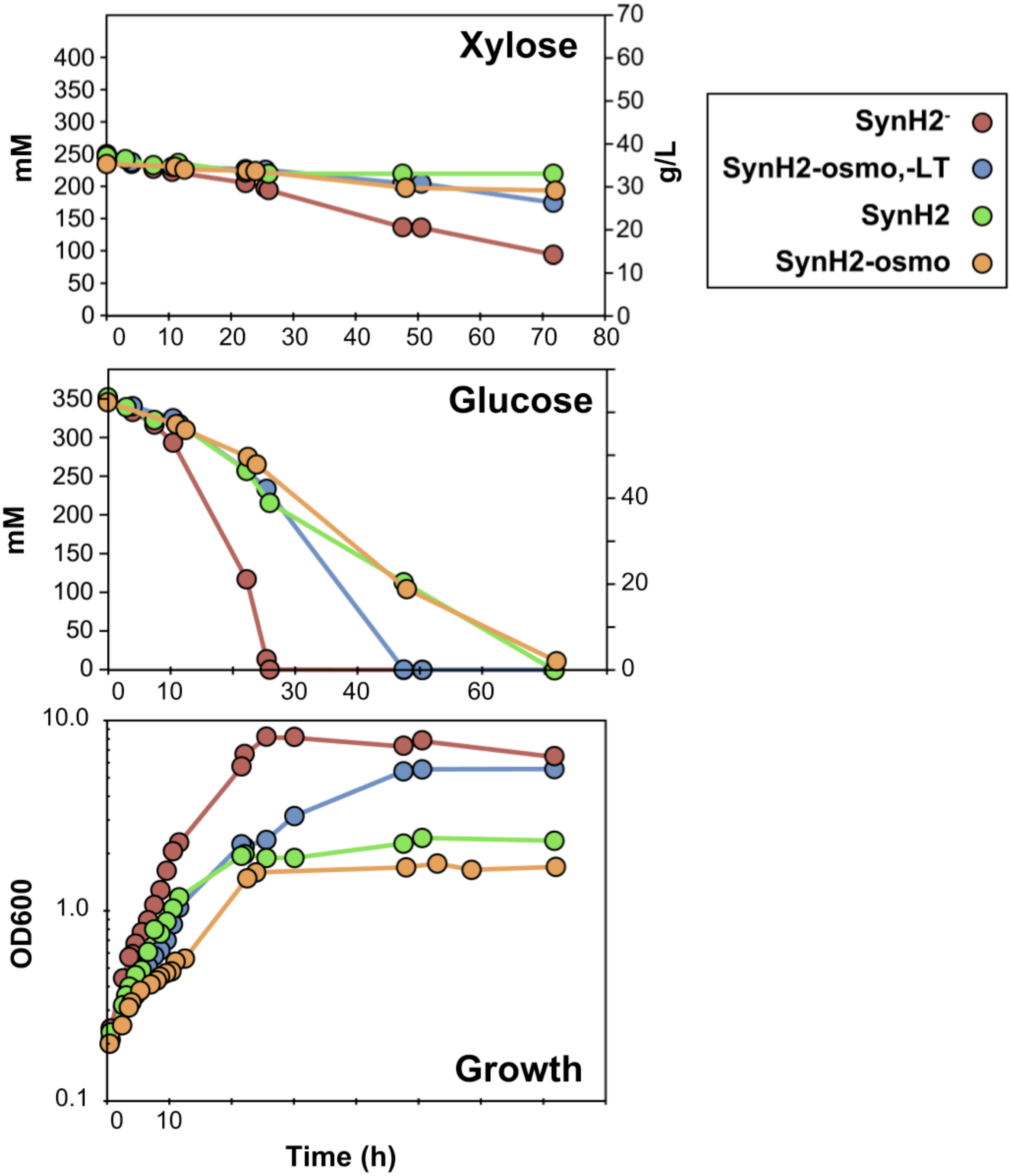

Figure S1. Effect of osmolytes and inhibitors on growth and sugar utilization in synthetic media.

GLBRCE1 was grown anaerobically in bioreactors in modified synthetic media. **SynH2**: second generation synthetic medium, **SynH2$^-$**: SynH2 lacking LC-derived inhibitors, **SynH2-osmo, -LT**: SynH2 lacking both osmolytes and inhibitors, **SynH2-osmo**: SynH2 lacking osmolytes but still containing inhibitors.
