# Supplementary material for: Aromatic inhibitors derived from ammonia-pretreated lignocellulose hinder bacterial ethanologenesis by activating regulatory circuits controlling inhibitor efflux and detoxification
Source: Front Microbiol. 2014 Aug 13;5:402. doi: 10.3389/fmicb.2014.00402 (PMC4132294; doi:10.3389/fmicb.2014.00402)
Supplement: Supplementary file 1 [file DataSheet1.ZIP › Figure S2.pdf]

Figure S2

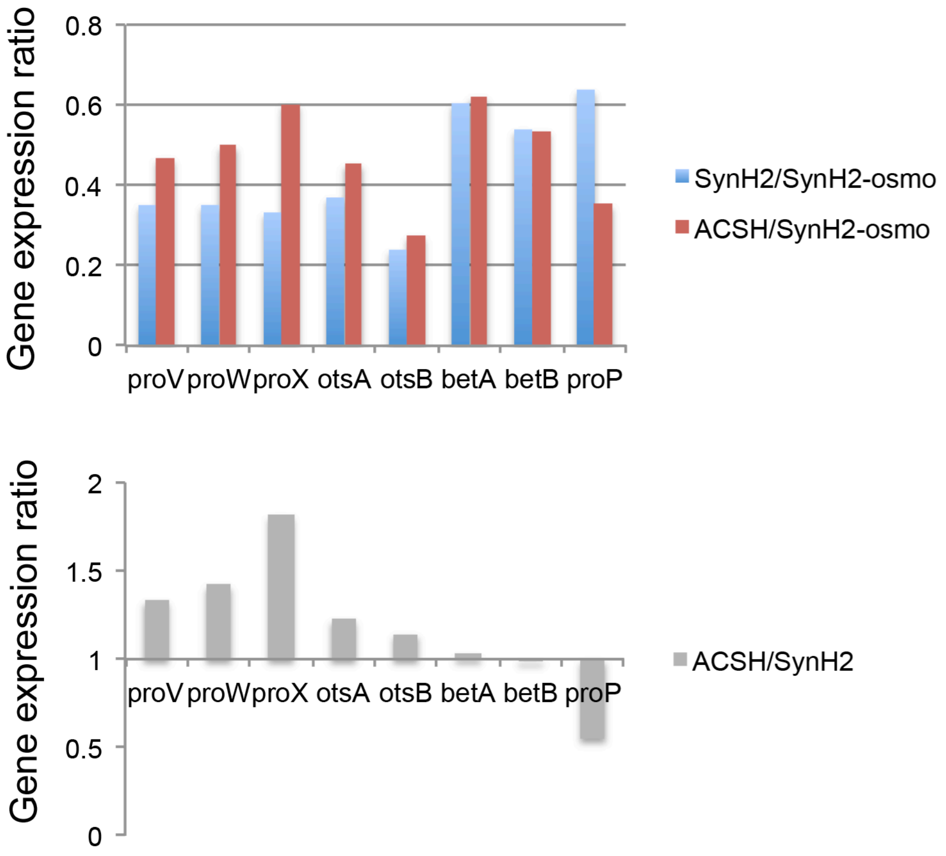

Figure S2. Effect of osmolytes on expression of osmotic stress genes.
RNA was prepared from log-phase cultures of GLBRCE1 grown anaerobically in bioreactors as shown in Figure S1 and hybridized to microarrays. Gene intensities were normalized and expression ratios calculated that compared cultures grown in media containing osmolytes to those grown in media lacking osmolytes. *Top panel*: gene expression ratios showing similar induction ratios for SynH2 and ACSH relative to SynH2 lacking osmolytes. *Bottom panel*: comparison of ACSH to SynH2. The ratio is expected to be equal to 1 if expression of osmoprotectant genes are equivalent in the two media.
