# Supplementary material for: Aromatic inhibitors derived from ammonia-pretreated lignocellulose hinder bacterial ethanologenesis by activating regulatory circuits controlling inhibitor efflux and detoxification
Source: Front Microbiol. 2014 Aug 13;5:402. doi: 10.3389/fmicb.2014.00402 (PMC4132294; doi:10.3389/fmicb.2014.00402)
Supplement: Supplementary file 1 [file DataSheet1.ZIP › Figure S3.pdf]

Figure S3

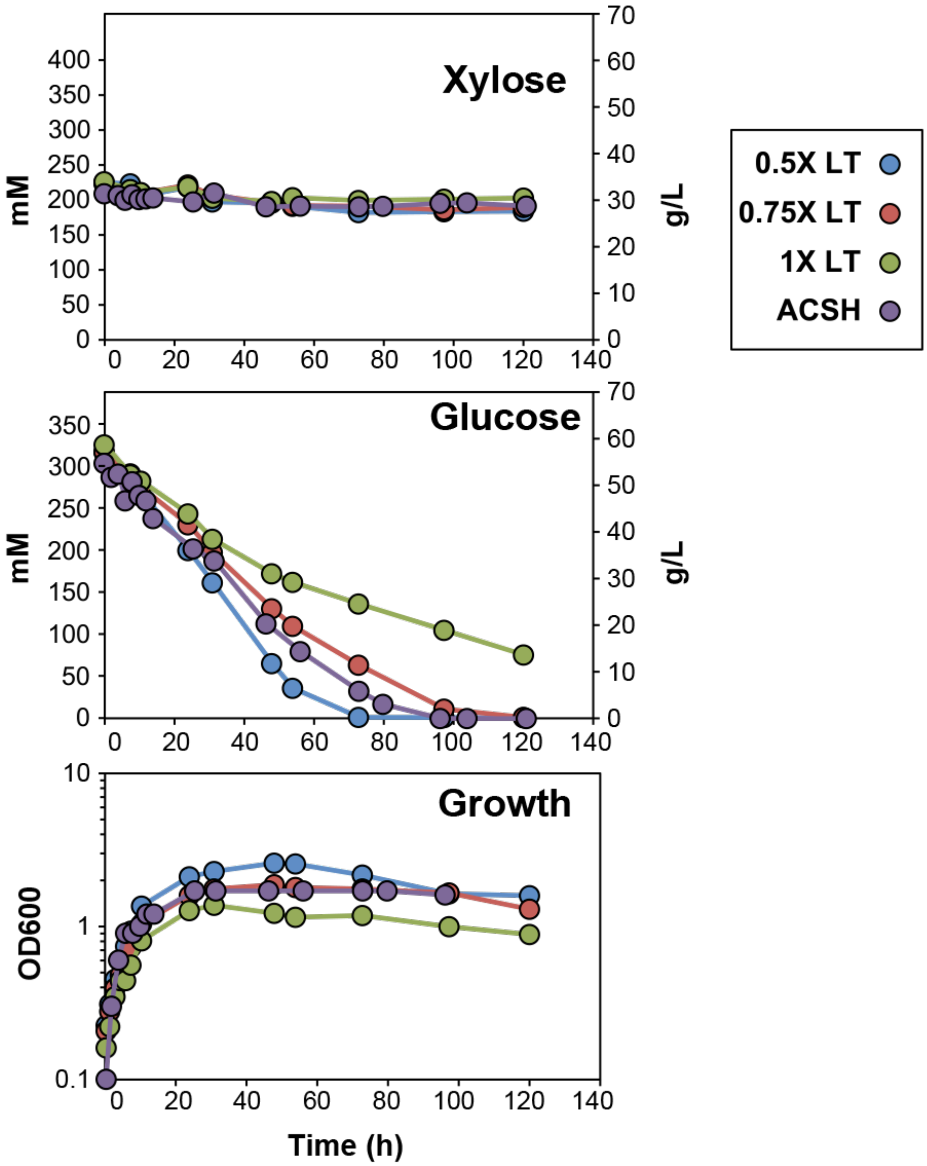

Figure S3. Determination of the LT concentration that mimics the growth and sugar utilization properties of ACSH. GLBRCE1 was grown in flasks in an anaerobic chamber in SynH2$^-$ supplemented with different concentrations of LTs. 1X LT is equivalent to the concentrations listed in ACSH in Table 1 for each of the 14 different inhibitors. GLBRCE1 grown in ACSH is shown as a reference.
