# Supplementary material for: Aromatic inhibitors derived from ammonia-pretreated lignocellulose hinder bacterial ethanologenesis by activating regulatory circuits controlling inhibitor efflux and detoxification
Source: Front Microbiol. 2014 Aug 13;5:402. doi: 10.3389/fmicb.2014.00402 (PMC4132294; doi:10.3389/fmicb.2014.00402)
Supplement: Supplementary file 1 [file DataSheet1.ZIP › Figure S4.pdf]

Figure S4

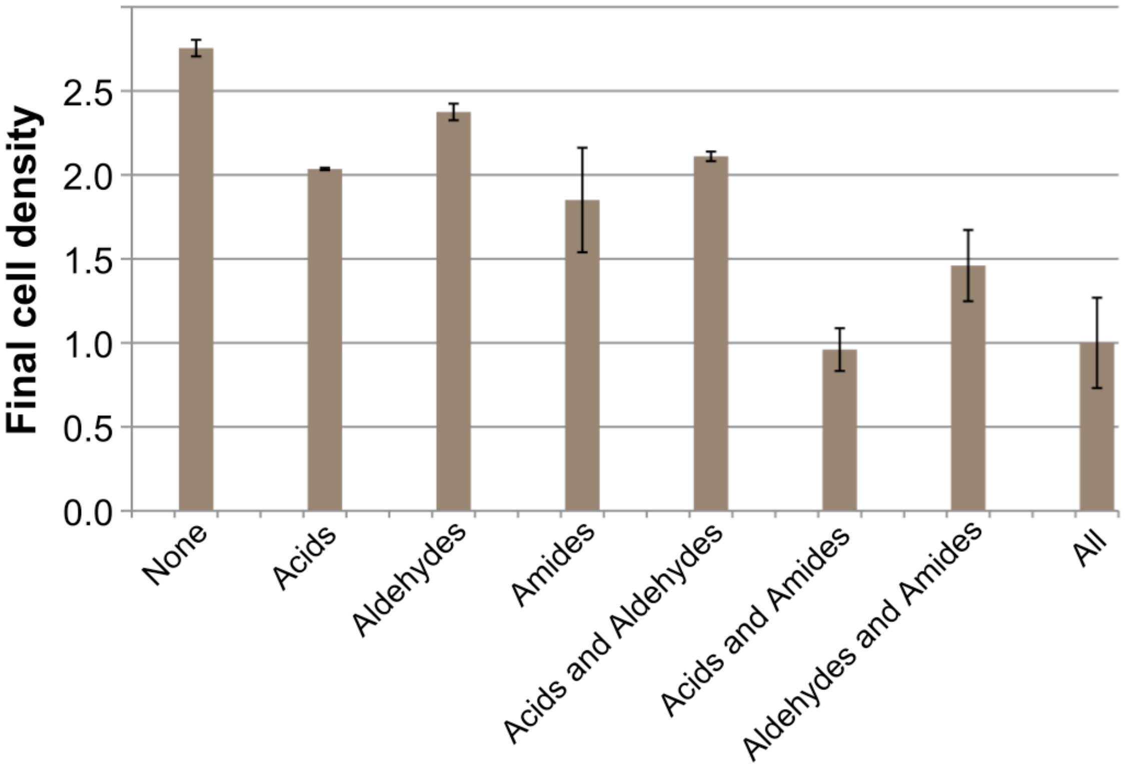

Figure S4. Effect of different inhibitor classes on growth in SynH2^{-}. GLBRCE1 was grown in flasks in an anaerobic chamber. The 14 inhibitors were separated into classes based on their functional group chemistry (Table 1) and added to SynH2^{-}. As a control, the strain was grown to stationary phase in unsupplemented SynH2^{-} (none). The final cell density for cultures is shown. “All” refers to the entire cocktail of inhibitors as shown in Table 1.
