# Supplementary material for: Aromatic inhibitors derived from ammonia-pretreated lignocellulose hinder bacterial ethanologenesis by activating regulatory circuits controlling inhibitor efflux and detoxification
Source: Front Microbiol. 2014 Aug 13;5:402. doi: 10.3389/fmicb.2014.00402 (PMC4132294; doi:10.3389/fmicb.2014.00402)
Supplement: Supplementary file 1 [file DataSheet1.ZIP › Figure S5.pdf]

Figure S5

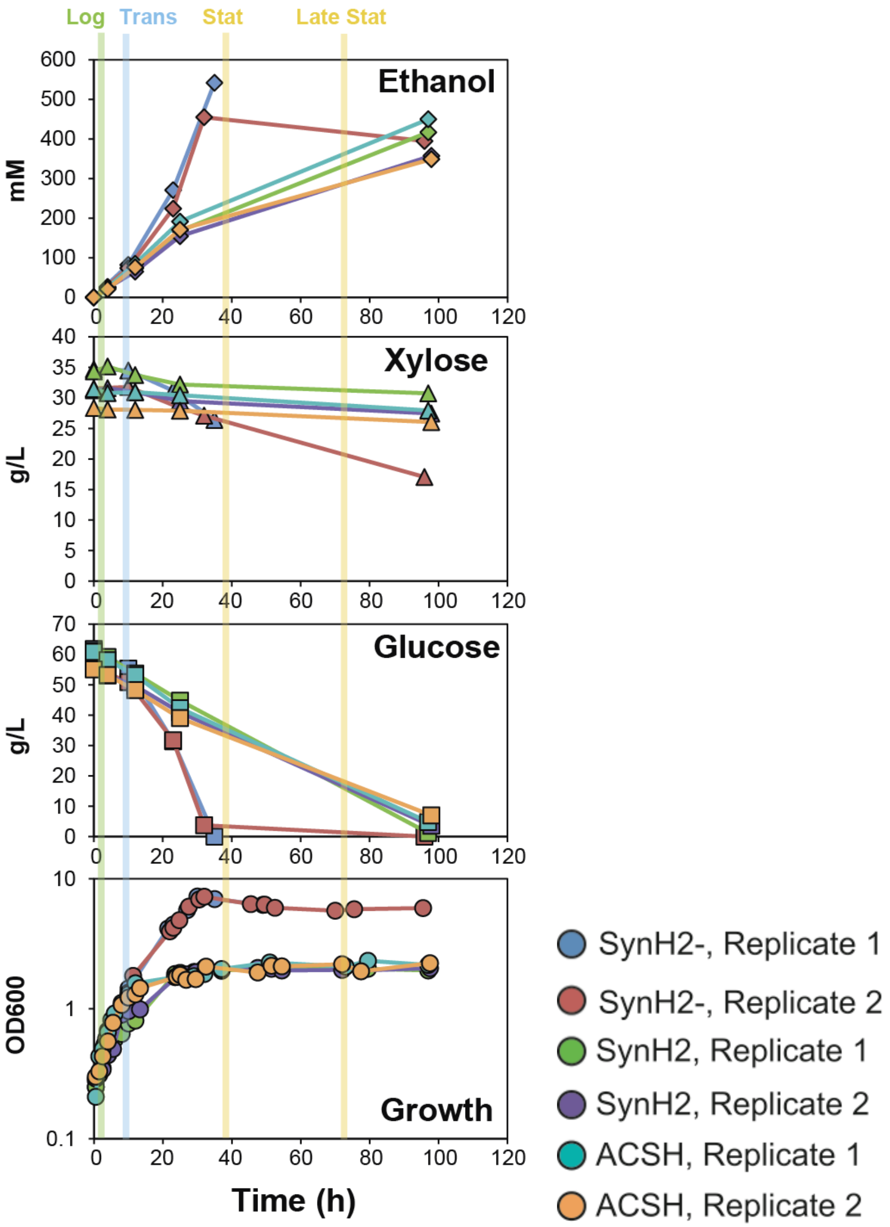

Figure S5. Replicate cultures, GLBRCE1 fermentations.
GLBRCE1 was grown anaerobically in bioreactors in SynH2⁻, SynH2, and ACSH. Growth (OD600), sugar utilization, and ethanol production were measured and plotted as a function of time. Cultures are replicates of the experiment shown in Figure 1 of the main text.
