# Supplementary material for: Aromatic inhibitors derived from ammonia-pretreated lignocellulose hinder bacterial ethanologenesis by activating regulatory circuits controlling inhibitor efflux and detoxification
Source: Front Microbiol. 2014 Aug 13;5:402. doi: 10.3389/fmicb.2014.00402 (PMC4132294; doi:10.3389/fmicb.2014.00402)
Supplement: Supplementary file 1 [file DataSheet1.ZIP › Supplemental Information.pdf]

Supplemental Information for

Aromatic inhibitors derived from ammonia pretreated lignocellulose hinder bacterial ethanologenesis by activating regulatory circuits controlling inhibitor efflux and detoxification

David H. Keating[1†], Yaoping Zhang[1†], Irene M. Ong[1†], Sean McIlwain[1†], Eduardo H. Morales[1,2†], Jeffrey A. Grass[1,3], Mary Tremaine[1], William Bothfeld[1], Alan Higbee[1], Arne Ulbrich[4], Allison Balloon[4], Michael S. Westphall[2,4], Josh Aldrich[5], Mary Lipton[5], Joonhoon Kim[6], Oleg Moskvin[1], Yury V. Bukhman[1], Joshua Coon[1,2,4], Patricia J. Kiley[1,2], Donna M. Bates[1]* and Robert Landick[1,3,7]*

[1] Great Lakes Bioenergy Research Center, University of Wisconsin-Madison, Madison, WI USA
[2] Department of Biomolecular Chemistry, University of Wisconsin-Madison, Madison, WI USA
[3] Department of Biochemistry, University of Wisconsin-Madison, Madison, WI USA
[4] Department of Chemistry, University of Wisconsin-Madison, Madison, WI USA
[5] Pacific Northwest National Laboratory, Richland, WA USA
[6] Department of Chemical and Biological Engineering, University of Wisconsin-Madison, Madison, WI USA
[7] Department of Bacteriology, University of Wisconsin-Madison, Madison, WI USA

CONTENTS:

Supplemental Results

Supplemental Methods

TABLE S1. Metabolite changes in response to inhibitors

TABLE S2 (xls). Gene expression levels measured by RNAseq.

TABLE S3. Pathways, transporters, and regulons whose genes exhibit discrepant changes in ACSH and SynH2 relative to SynH2^{-}.

TABLE S4. Pathways, transporters, and regulons whose genes exhibit consistent changes in ACSH and SynH2 relative to SynH2^{-}.

TABLE S5 Pathways, transporters, and regulons involved in N regulation whose genes exhibit consistent changes in ACSH and SynH2 relative to SynH2.

TABLE S6. (xls). Relative protein levels measured by iTraq in ACSH, SynH2, and SynH2^{-} cells.

TABLE S7 (xls). Discrepant Protein/RNA ratios between SynH2 and SynH2^{-} cells in exponential (A), transition (B), and stationary (C) phases. Peptide counts for protein expression levels (D). Peptide identity features (E).

TABLE S8. LC-derived inhibitor profile changes during fermentation

TABLE S9 (xls). RNAseq statistical analysis

FIGURE S1 Effects of osmolytes and inhibitors on growth in SynH.

FIGURE S2. Effects of osmolytes on osmoprotectant gene expression.

FIGURE S3. Effects of different inhibitor concentrations on growth in SynH.

FIGURE S4. Effects of different inhibitor classes on growth in SynH.

FIGURE S5. Biological replicates of cell growth profiles in ACSH, SynH2, and SynH2^{-}.

Supplemental Results

Previous studies of the GLBRCE1 ethanologen in SynH1, our first generation synthetic medium, suggested that ACSH contained protective osmolytes because osmotic stress genes were induced in SynH1 relative to ACSH (Schwalbach et al., 2012). Analysis of ACSH identified compounds that could function in osmoprotection, including betaine, choline, and carnitine (Table 1). To determine whether inclusion of the osmoprotectants and inhibitors could account for the major differences between SynH1 and ACSH, we first modified the base recipe of SynH1 by inclusion of 14 compounds present in ACSH (**Table 1**; galactose, mannose, fructose, lactate, pyruvate, citrate, nitrate, formate, malate, succinate, acetate, acetamide, glycerol, and D-arabinose in place of L-arabinose to avoid repression of *xyl* genes). This reformulation yielded a SynH that lacked osmolytes and aromatic inhibitors (SynH2-osmo, -LT, where LT denotes lignotoxins, an alternative name for LC-derived inhibitors), a SynH that included osmolytes (betaine, choline, and carnitine) but not LC-derived inhibitors ($SynH2^{-}$), a SynH that included the 14 aromatic inhibitors present in ACSH but not the osmolytes (SynH2-osmo), and a complete SynH that included both osmolytes and aromatic inhibitors (SynH2). In SynH2-osmo, -LT (lacking protective osmolytes and inhibitors), cell growth ceased with more than half the glucose remaining (**Figure S1**; blue lines and symbols), consistent with prior observations (Schwalbach et al., 2012). A second phase of growth then occurred, which may correspond to accumulation of synthesized proline (Schwalbach et al., 2012). Inclusion of betaine, choline, and carnitine at concentrations equivalent to those in ACSH ($SynH2^{-}$, red lines and symbols) allowed cell growth to continue until glucose was exhausted; $SynH2^{-}$ also allowed increased xylose consumption in stationary phase, consistent with observations in minimal medium (Underwood et al., 2004; Miller and Ingram, 2007). Inclusion of betaine, choline, and carnitine in SynH2 also eliminated the elevated expression of osmotic stress genes relative to ACSH that had been observed in SynH1 (**Figure S2**) (Schwalbach et al., 2012). We concluded that inclusion of the protective osmolytes betaine, choline, and carnitine in the SynH2 recipe improved the replication of cell physiology of *E. coli* growing in ACSH.

Although cells grew robustly in $SynH2^{-}$, addition of LC-derived aromatic inhibitors reversed this effect (**Figure S1**; SynH2, green lines and symbols). At the concentrations measured in ACSH

(Table 1), the inhibitors slowed growth and glucose consumption to rates less than observed in ACSH **(Figure S3)**. Therefore, we tested SynH compositions containing reduced concentrations of the 5 major inhibitors present in the recipe (coumaric acid, ferulic acid, coumaroyl amide, feruloyl amide, and HMF). We found that 0.75X and 0.5X concentrations of these inhibitors more closely mimicked the growth and sugar consumption profiles observed in ACSH (**Figure S3**). These preliminary inhibitor studies also revealed that when tested as groups of phenolic carboxylates, phenolic amides, and aromatic aldehydes at the concentrations found in ACSH, the strongest inhibition was caused by the phenolic carboxylates and amides with somewhat lesser effects of the aldehydes (**Figure S4**). Strikingly, the phenolic carboxylates and amides combined were just as inhibitory as the ternary combination of carboxylates, amides, and aldehydes.

Taken together, these results allowed us to define a recipe for SynH2 in which inclusion of LC-derived aromatic inhibitors slowed growth and inhibited sugar conversion to rates equivalent to those observed in ACSH (**Table 1**). SynH2 is a close match to the chemical composition of ACSH, except that the five most abundant aromatic inhibitors (HMF, ferulic acid, coumaric acid, feruloyl and coumaroyl amides) are present at about half the levels typically measured for ACSH (Table 1). These results may suggest that as-yet unidentified ACSH components allow greater inhibitor tolerance. More importantly for the purpose of the present study, they enabled our immediate goal of investigating the effect of the inhibitors on gene expression and cell physiology with confidence that the results would be relevant to authentic hydrolysates derived from ammonia-pretreated biomass.

Supplemental Methods

Growth of *E. coli* in flasks to determine the effect of different classes of inhibitors.

GLBRCE1 was grown in LB medium aerobically, and transferred into $SynH2^{-}$ medium with 1:10 dilution, and then grew in anaerobic chamber for overnight. The overnight cultures were inoculated into 25 ml of $SynH2^{-}$ (Table 1, pre-incubated in anaerobic chamber) with different classes of inhibitors with initial OD at 0.2. Cells were then grown at $37^{o}C$ on a stirrer plate in anaerobic

chamber with two biological replicates, and OD_{600} was measured after 24 h incubation. All media contained 10 μg/ml gentamicin to maintain pPET plasmid.

Microarray gene expression analysis.

Samples for microarray transcriptomic analysis were captured and RNA extracted as described previously (Schwalbach et al., 2012). Transcript levels measured by microarrays were carried out as described previously (Schwalbach et al., 2012). Signal intensities were pre-processed by robust multichip averaging (RMA) and gene expression signals were quantile-normalized using the Partek software package.

Measurement of metabolites by reverse-phase, ion-pairing HPLC

Preparation of intracellular extracts. 20 ml of cell culture was rapidly removed from bioreactors with a 50 ml sterile syringe and 4 ml aliquots were applied to a filtration manifold unit (Hoefer FH 225V) outfitted with sterile 25 ml nylon filters (Whatman; Nylon; 0.45um pore size), and the cells captured on the filters under vacuum. To reduce the background associated with metabolites present in ACSH and SynH the cells were then rapidly washed with 5 ml of M9 medium (Neidhardt et al., 1974) lacking a carbon source. The filters were then removed and rapidly placed in 15 ml conical tubes containing extraction buffer (acetonitrile:methanol:water 40:40:20 by vol plus 0.1% formic acid) (Amador-Noguez et al., 2011) and flash frozen in a dry ice ethanol bath.

Reverse Phase Ion Pairing HPLC was carried out by an adaptation of the method of Buescher et. al (Buescher et al., 2010), and was used to quantify reduced and oxidized glutathione in intracellular extracts. Compounds were separated on a Waters Acquity Ascentis HSS-T3 C18 column, 150x2.0mm, 1.8 μm particle size. Mobile phase A consisted of 95:5 Water:methanol + 10mM tributylamine (TBA) + 15mM acetic acid, and mobile phase B was isopropyl alcohol.

ESI-MS/MS was carried out on an Agilent 6460 triple quadrupole mass spectrometer. The source gas was nitrogen supplied by a Cole Parmer N2-14A-K727 nitrogen generator and the collision cell gas was >99% pure compressed nitrogen. ESI source conditions were: Gas Temp 300 °C, Gas Flow 11mL / min, Nebulizer 45psi, Sheath Gas Temperature 360^0C, Sheath Gas Flow 11 mL/min, Capillary (-)2000V, Nozzle 1000V. Detection was achieved by negative ionization mode multireaction monitoring (MRM). The sodium hydroxide in the *HPAEC* eluent was removed with

Thermo Dionex ASRS 300 2mm RFIC self-regenerating suppressor powered by a RFIC Reagent Free controller set to supply 100mA.

References

Amador-Noguez, D., Brasg, I.A., Feng, X.J., Roquet, N., and Rabinowitz, J.D. (2011). Metabolome remodeling during the acidogenic-solventogenic transition in Clostridium acetobutylicum. *Appl Environ Microbiol* 77**,** 7984-7997.

Buescher, J.M., Moco, S., Sauer, U., and Zamboni, N. (2010). Ultrahigh performance liquid chromatography-tandem mass spectrometry method for fast and robust quantification of anionic and aromatic metabolites. *Anal Chem* 82**,** 4403-4412.

Miller, E.N., and Ingram, L.O. (2007). Combined effect of betaine and trehalose on osmotic tolerance of *Escherichia coli* in mineral salts medium. *Biotechnol Lett* 29**,** 213-217.

Neidhardt, F.C., Bloch, P.L., and Smith, D.F. (1974). Culture medium for enterobacteria. *J Bacteriol* 119**,** 736-747.

Schwalbach, M.S., Keating, D.H., Tremaine, M., Marner, W.D., Zhang, Y., Bothfeld, W., Higbee, A., Grass, J.A., Cotten, C., Reed, J.L., Da Costa Sousa, L., Jin, M., Balan, V., Ellinger, J., Dale, B., Kiley, P.J., and Landick, R. (2012). Complex physiology and compound stress responses during fermentation of alkali-pretreated corn stover hydrolysate by an Escherichia coli ethanologen. *Appl Environ Microbiol* 78**,** 3442-3457.

Underwood, S.A., Buszko, M.L., Shanmugam, K.T., and Ingram, L.O. (2004). Lack of protective osmolytes limits final cell density and volumetric productivity of ethanologenic *Escherichia coli* KO11 during xylose fermentation. *Appl Environ Microbiol* 70**,** 2734-2740.
