# Supplementary material for: Aromatic inhibitors derived from ammonia-pretreated lignocellulose hinder bacterial ethanologenesis by activating regulatory circuits controlling inhibitor efflux and detoxification
Source: Front Microbiol. 2014 Aug 13;5:402. doi: 10.3389/fmicb.2014.00402 (PMC4132294; doi:10.3389/fmicb.2014.00402)
Supplement: Supplementary file 1 [file DataSheet1.ZIP › Table S1.pdf]

Table S1. Select Metabolite Concentration in GLBRCE1 During Growth in SynH2^{-} and SynH2.

**Metabolite (mM)**	**SynH2^{-} Exp**	**SynH2 Exp**	**SynH2^{-} Trans**	**SynH2 Trans**	**SynH2^{-} Stat**	**SynH2 Stat**
Glucose-6-phosphate	4 ± 4	1.6 ± 1.2	2 ± 2	0.26 ±0.04	2 ± 3	0.8 ± 0.7
Fructose-6-phosphate	1.1 ± 0.1	1.1 ± 0.7	0.7 ± 0.5	0.5 ± 0.4	0.6 ± 0.5	1.5 ± 1.1
Fructose-1,6-diphosphate	80 ± 10	11 ± 4	70 ± 40	4 ± 4	60 ± 60	10 ± 4
2- & 3-Phosphoglyceric acid	11 ± 3	5 ± 2	8 ± 4	2 ± 1	6 ± 5	3 ± 0.2
Phosphoenolpyruvic acid	0.4 ± 0.2	0.5 ± 0.5	0.3 ± 0.1	0.3 ± 0.3	0.3 ± 0.2	0.2 ± 0.1
Pyruvic acid	0.3 ± 0.5	40 ± 20	0.3 ± 0.3	3 ± 0.8	0.3 ± 0.3	0.1 ± 0.1
Citric acid	12 ± 14	100 ± 60	5 ± 5	40 ± 6	5 ± 6	4 ± 4
2-ketoglutaric acid	0.1 ± 0.1	100 ± 70	0.7 ± 0.4	1 ± 1	0.4 ± 0.1	1.1 ± 0.5
ATP	4.7 ± 0.8	3.3 ± 0.5	ND	ND	2 ± 2	0.9 ± 0.3
Glutathione (reduced)	9 ± 3	0.4 ± 0.1	3 ± 3	1 ± 0.4	0.2 ± 0.2	0.004 ± 0.004
Glutathione (oxidized)	0.2 ± 0.2	ND	0.3 ± 0.3	0.07 ± 0.07	0.06 ± 0.07	ND
NADH/NAD	1.1 ± 0.1	0.4 ± 0.1	ND	ND	ND	ND
NADPH/NADP	1.5 ± 0.2	0.8 ± 0.2	ND	ND	ND	ND

ND= Below limit of detection.
Errors are s.d. of experimental triplicates including error propagated from standard curves.
