# Supplementary material for: Aromatic inhibitors derived from ammonia-pretreated lignocellulose hinder bacterial ethanologenesis by activating regulatory circuits controlling inhibitor efflux and detoxification
Source: Front Microbiol. 2014 Aug 13;5:402. doi: 10.3389/fmicb.2014.00402 (PMC4132294; doi:10.3389/fmicb.2014.00402)
Supplement: Supplementary file 1 [file DataSheet1.ZIP › Table S3.pdf]

Table S3. Pathways, transporters, and regulons whose genes exhibit discrepant changes in ACSH and SynH2 relative to SynH2$^-$

	Exp	Exp	Tran	Tran	Stat	Stat	Genes
Pathways	***SynH2[a]***	***ACSH[a]***	***SynH2[a]***	***ACSH[a]***	***SynH2[a]***	***ACSH[a]***	
Carbon/Energy Source							
Sorbitol catabolism	0.48	0.55	0.55	17.52	0.21	4.10	srlD
Mannitol degradation	1.27	5.14	0.69	0.79	0.58	0.39	mtlD
Citrate lyase	0.88	1.72	319.24	3.44	12.72	0.24	citX citG citD citF citE
D-malate catabolism	0.09	0.00	33.59	1.09	25.54	0.48	dmlA
L-arabinose catabolism	1.49	52.93	1.23	11.46	0.16	0.35	araA araD araB
Amino acid biosynthesis							
Tryptophan biosynthesis	0.84	6.38	0.11	0.37	1.00	0.58	trpC trpE trpD trpA trpB
Asparagine biosynthesis	0.17[b]	1.07	0.60	9.82	0.83	0.65	asnB asnA
Ornithine biosynthesis	0.44	1.39	0.04	0.04	0.09	3.25	argA argB argC argD argE
Cofactor biosynthesis							
Thiamine diphosphate biosynthesis	1.21	5.70	0.99	3.11	0.65	2.17	thiD thiC thiL thiE thiG dxs thiF thiH csdA sufS iscS thiI thiS
Biotin Biosynthesis	2.33	0.70	1.34	0.51	0.68	0.43	bioH fabB fabI fabZ fabG bioC bioF bioD bioB bioA acpP
Deoxyribo-nucleoside catabolism	4.70	1.82	2.33	0.57	0.36	0.29	deoD add deoB deoC adhE mhpF cdd deoA
Regulatory/Stress response							
Fur	0.89	0.70	0.89	14.79	16.05	0.98	nac amtB ftnA

Nitric oxide stress	1.72	6.50	0.94	0.81	0.68	0.42	hmp hcr hcp nirB
	**Exp**	**Exp**	**Tran**	**Tran**	**Stat**	**Stat**	**Genes**
Transporters	***SynH2***[a]	***ACSH***[a]	***SynH2***[a]	***ACSH***[a]	***SynH2***[a]	***ACSH***[a]	
Carbohydrates							
Arabinose-proton symport	0.92	8.03	0.88	2.47	1.88	2.61	araE
Citrate-succinate antiporter	0.34	1.14	51.94	1.38	7.62	0.90	citT
Sorbitol PTS	0.50	0.86	0.47	20.24	0.17	8.53	srlE srlB srlA
Amino acids							
High-affinity tryptophan transport	0.77	12.79	0.59	0.79	2.51	0.11	mtr
Peptide transport	0.85	0.42	6.26	0.95	4.62	1.36	dtpA
Metal efflux							
Copper/silver efflux	0.87	13.29	0.80	1.88	0.55	0.39	cusA cusF cusB cusC
	**Exp**	**Exp**	**Tran**	**Tran**	**Stat**	**Stat**	**Genes**
Regulons	***SynH2***[a]	***ACSH***[a]	***SynH2***[a]	***ACSH***[a]	***SynH2***[a]	***ACSH***[a]	
AraC	1.45	50.55	1.20	11.11	0.19	0.57	araD araB araA araE
BirA	5.03	0.52	1.37	0.29	0.27	0.15	bioB bioD bioA bioC bioF
DpiA	0.90	1.34	81.96	1.93	9.54	0.35	citX dpiB dpiA exuT citC citF citE mdh citD citG appY
OxyR	1.73	4.96	1.72	3.48	1.69	4.22	ybjC hcp nfsA rimK hcr trxC grxA ybjN uxuA hemH fur
SrlR	0.50	0.98	0.55	13.18	0.26	2.26	gutQ gutM srlR srlD srlB srlE srlA

[a]Values represent median fold changes for the gene set; Blocks in bold indicate significant fold-changes with an aggregate p-value ≤0.05.
[b]p-value = 0.09.
