# Supplementary material for: Aromatic inhibitors derived from ammonia-pretreated lignocellulose hinder bacterial ethanologenesis by activating regulatory circuits controlling inhibitor efflux and detoxification
Source: Front Microbiol. 2014 Aug 13;5:402. doi: 10.3389/fmicb.2014.00402 (PMC4132294; doi:10.3389/fmicb.2014.00402)
Supplement: Supplementary file 1 [file DataSheet1.ZIP › Table S4.pdf]

Table S4. Pathways, transporters, and regulons whose genes exhibit consistent changes in ACSH and SynH2 relative to SynH2⁻

	Exp	Exp	Tran	Tran	Stat	Stat	Genes
Pathways	*SynH2[a]*	*ACSH[a]*	*SynH2[a]*	*ACSH[a]*	*SynH2[a]*	*ACSH[a]*	
Carbon/Energy Source							
Xylose metabolism	0.20	0.20	0.09	0.07	0.19	0.15	xylR xylB xylH xylA xylE xylF xylG
Amino acid metabolism							
Cysteine biosynthesis and S assimilation	1.63	1.31	0.20	0.31	0.92	0.91	cysH cysJ cysI cysN cysD cysC cysE cysK cysM serC serA serB aphA trxA trxC cysQ metC malY metB metA
Methionine biosynthesis	0.33	0.58	0.20	0.23	3.30	0.46	metA metE metH metC malY metB
Ornithine degradation	1.11	0.83	0.85	0.78	0.26	0.16	speF prr patD patA puuD puuC puuB puuA
Cofactor biosynthesis							
Superpath-way of S-adenosyl-L-methionine biosynthesis	0.50	0.64	0.20	0.26	1.32	0.44	metA metE metH metC malY metB lysC asd thrA metL metK
Phosphonate and phosphinate metabolism	1.78	1.18	1.18	0.98	0.04	0.04	yjdM yncA phnI phnG phnH phnL phnM phnJ phnP
Purine Deoxyribo-nucleotide synthesis	2.27	1.54	2.48	2.73	1.29	0.94	nrdA nrdB nrdE nrdF ndk nrdD fldA fldB nrdH trxA trxC
Pyrimidine synthesis	0.14	0.44	1.01	0.40	0.84	0.78	carB carA pyrB pyrI pyrC pyrE pyrF pyrD pyrH ndk pyrG
Pyruvate dehydro-genase	4.42	5.61	0.27	1.24	0.25	0.61	lpd aceE aceF

Glutathione synthesis, reduction and transport	2.07[b]	1.69	1.42	1.66	1.36	2.31	gshA gshB gor
Detoxification/Stress response							
Aldehyde detoxifica-tion	31.87	7.15	4.04	0.77	0.32	0.75	frmB yeiG frmA yqhD dkgA
Glutamate dependent acid resistance	0.03	0.03	0.48	0.71	4.30	0.29	gadA gadB
Macromolecules							
Flagellar assembly	0.31	0.24	0.89	0.71	0.51	0.44	fliT flgN flhA flhC motB flgL fliS flhD fliR flgK flgM flgA motA fliC fliD flhB fliP fliQ fliK fliH flgI fliI fliJ flgH flgG fliG fliN fliE fliM fliO flgF fliF flgE flgD flgC flgB fliK
	**Exp**	**Exp**	**Tran**	**Tran**	**Stat**	**Stat**	Genes
Transporters	*SynH2*[a]	*ACSH*[a]	*SynH2*[a]	*ACSH*[a]	*SynH2*[a]	*ACSH*[a]	
Carbohydrates							
Galactitol PTS	0.10	0.21	0.12	0.05	0.16	0.05	gatA gatB gatC
Maltose ABC transporter	0.36	0.47	0.17	0.10	0.75	0.15	malE malG malF malK
Mannose transport	0.41	0.84	0.34	0.16	0.31	0.10	manX manY manZ
Xylose transport	0.17	0.20	0.07	0.06	0.23	0.15	xylF xylH xylG
Amino acids and peptides							
Arginine ABC transporter	0.98	1.48	0.19	0.20	0.26	2.31	argT hisM hisP hisQ artJ artI artQ artM artP
Glutamine ABC transporter	0.16	0.19	6.63	12.35	8.88	3.90	glnH glnP glnQ

Methionine ABC transporter	0.12	0.53	0.19	0.13	3.15	0.19	metQ metI metN
Ser-Thr/Na+ symport	0.25	0.06	0.19	0.19	0.75	0.14	sstT
Dipeptide ABC transporter	0.16	0.16	0.21	0.57	0.51	0.20	dppA dppC dppB dppF dppD
Murein tripeptide ABC transporter	0.17	0.17	0.27	0.19	0.41	0.33	mppA oppF oppD oppC oppB
Metal ions and cofactors							
Ferric enterobactin ABC transporter	1.00	0.81	1.13	0.68	0.06	0.12	fepB fepG fepD fepC
Molybdate ABC transporter	0.92	0.87	0.13	0.08	0.82	0.10	modA modB modC
Nickel ABC transporter	2.23	1.02	0.58	0.28	0.13	0.09	nikA nikC nikB nikE nikD mgtA
Selenite ABC Transporter	33.12	13.45	0.08	0.07	0.35	0.93	sbp cysP cysU cysW cysA
Nicotinamide ribonulceo-side uptake permease	0.21	0.06	0.17	0.08	2.10	0.17	pnuC
Efflux pumps							
Purine ribonucleo-side efflux	5.69	2.78	2.27	1.49	0.49	0.20	nepI
RND-PET efflux pumps	3.59	4.06	1.71	2.43	1.19	1.75	aaeA aaeB acrA acrE acrB
Other transport functions							
Solute transport	0.26	0.58	0.27	0.89	0.22	0.34	ompG
	**Exp**	**Exp**	**Tran**	**Tran**	**Stat**	**Stat**	Genes
Regulons	*SynH2*[a]	*ACSH*[a]	*SynH2*[a]	*ACSH*[a]	*SynH2*[a]	*ACSH*[a]	
AaeR	12.50	5.40	8.75	9.35	8.71	13.53	aaeX aaeA aaeB
AdiY	0.03	0.03	0.50	0.74	4.74	0.44	gadX gadB gadC gadA

AppY	0.13	0.23	0.40	0.77	0.47	0.16	appA appB appC hyaD hyaE hyaF hyaA hyaC hyaB
CaiF	1.26	1.15	0.87	0.71	0.20	0.12	caiE caiD caiC caiB caiA caiT fixX fixC fixB fixA
CynR	1.11	1.14	1.30	0.68	0.19	0.12	cynR cynT cynS cynX cynR cynT cynS cynX
CysB	26.09	11.27	0.08	0.11	0.39	1.07	cysJ cysD cysI cysH cysC cysN cysP cysA cysU cysW cbl cysK tauA cysM tauB cysK cysB tauD tauC
DeoR	4.70	1.82	3.91	0.57	0.36	0.31	tsx napG deoC deoA deoB deoD
FecI	1.29	0.90	1.27	0.70	0.21	0.14	fecE fecD fecC fecB fecA fecR fecI
FhlA	1.42	1.40	1.25	0.80	0.24	0.14	hyfJ hyfI hyfH hyfG hyfF hyfE hyfD hyfC hyfB hyfA fdhF hypA hypB hypC hypD hypE hypF hydN hycH hycG hycF hycE hycD hycC hycB hycA hyfJ hyfI hyfH hyfG hyfF hyfE hyfD hyfC hyfB hyfA fdhF hypA hypB hypC hypD hypE hypF hydN hycH hycG hycF hycE hycD hycC hycB hycA
FliZ	0.13	0.16	0.68	1.05	1.75	1.07	yjbJ csgG flhC csgD flhD csgE csgA hdeA hdeB yhiD mdtF gadE mdtE gadB gadC
FrmR	217.77	23.53	34.76	0.27	0.25	0.99	frmR frmA frmB
GadX	0.09	0.22	1.15	3.45	5.34	1.75	amtB gadE hns gadX hdeB gadB hdeA
GatR1/R2	0.12	0.21	0.14	0.06	0.17	0.05	gatY gatZ gatC gatA gatD gatB

HyfR	1.11	0.91	1.08	0.85	0.24	0.14	hyfJ hyfI hyfH hyfG hyfF hyfE hyfD hyfC hyfB hyfA
lacI	0.17	0.14	0.25	0.14	0.83	0.25	lacZ lacY lacA
MalT	0.47	0.52	0.26	0.17	0.80	0.19	malQ malS malP malZ malM malG malF lamB malE malK
MetJ	0.16	0.51	0.20	0.18	3.37	0.32	ahpC metL folE metB yeiB metK metI metQ metC metR metN metA metF metE
MetR	0.17	0.35	0.15	0.25	4.77	0.22	metE metR glyA
MhpR	1.23	1.44	0.84	0.92	0.14	0.15	mhpE mhpF mhpD mhpA mhpC mhpB
Mlc	0.42	0.77	0.36	0.21	0.39	0.14	ptsH ptsI crr ptsG malT dgsA manX manY manZ
NadR	0.19	0.04	0.14	0.05	1.93	0.15	nadA pncB pnuC nadB
NarP	1.19	1.50	1.10	0.78	0.38	0.18	hcr hcp norW norV hyaA hyaB hyaC hyaD hyaE hyaF ccmH ccmG ccmF ccmE ccmD ccmC ccmB ccmA napC napB napH napG napA napD napF hcr hcp norW norV hyaA hyaB hyaC hyaD hyaE hyaF ccmH ccmG ccmF ccmE ccmD ccmC ccmB ccmA napC napB napH napG napA napD napF

PhoB	1.46	1.09	0.98	1.07	0.13	0.11	ugpQ ugpC ugpE ugpA ugpB psiE asr argP phoH phoU pstB pstA pstC pstS phnP phnO phnN phnM phnL phnK phnJ phnI phnH phnG phnF NA phnE phnD phnC phoB phoR phoA psiF phoE
PspF	0.20	0.18	0.26	0.28	0.42	0.80	pspG pspA pspB pspC pspD pspE
MarA/SoxS/ Rob	1.99	3.41	2.40	2.54	2.26	3.20	rob marR marA marB ybhT fumC acnA ybjN rimK ybjC ygiC ygiB zwf nfsA nfsB nfo micF inaA sodA tolC acrA acrB
TrpR	0.88	4.31	0.18	0.39	0.98	0.57	aroH mtr aroL yaiA aroM trpA trpB trpC trpD trpE
XylR	0.23	0.22	0.10	0.08	0.21	0.16	xylR xylB xylH xylA xylF xylG
YiaJ	1.40	1.36	1.13	0.66	0.15	0.21	lyxK yiaK yiaL yiaN yiaO
YqhC	20.78	6.26	3.50	1.07	0.41	0.60	yqhD dkgA

[a]Values represent median fold changes for the gene set; Blocks in bold indicate significant fold-changes with an aggregate p-value ≤0.05.

[b]p-value=0.08

[c]p-value=0.07

[c]p-value=0.08
