# Supplementary material for: Aromatic inhibitors derived from ammonia-pretreated lignocellulose hinder bacterial ethanologenesis by activating regulatory circuits controlling inhibitor efflux and detoxification
Source: Front Microbiol. 2014 Aug 13;5:402. doi: 10.3389/fmicb.2014.00402 (PMC4132294; doi:10.3389/fmicb.2014.00402)
Supplement: Supplementary file 1 [file DataSheet1.ZIP › Table S5.pdf]

Table S5. Pathways, transporters, and regulons involved in N regulation whose genes exhibit consistent changes in ACSH and SynH2 relative to SynH2^{-}

	**Exp**	**Exp**	**Tran**	Tran	Stat	**Stat**	**Genes**
Pathways	***SynH2***[a]	***ACSH***[a]	***SynH2***[a]	***ACSH***[a]	***SynH2***[a]	***ACSH***[a]	
Arginine catabolism	1.10	0.66	0.95	8.91	5.57	0.19	astA astB astC astD astE
	**Exp**	**Exp**	**Tran**	Tran	Stat	**Stat**	**Genes**
Transporters	***SynH2***[a]	***ACSH***[a]	***SynH2***[a]	***ACSH***[a]	***SynH2***[a]	***ACSH***[a]	
Ammonium/ ammonia transport	0.89	0.70	0.89	16.78	16.05	0.86	amtB
Regulons	***SynH2***[a]	***ACSH***[a]	***SynH2***[a]	***ACSH***[a]	***SynH2***[a]	***ACSH***[a]	
GlnG	0.85	0.57	0.95	13.49	11.79	2.82	nac glnA cbl yeaG glnH amtB argT astC glnP yeaH glnQ astA glnG glnL astD astB
RpoN	0.81	0.51	1.02	10.08	9.11	1.86	nac glnA yeaG glnH amtB argT astC glnP yeaH glnQ gltI yebV astA glnG glnL astD chaC astB zapB chaC

[a]Values represent median fold changes for the gene set; Blocks in bold indicate significant fold-changes with an aggregate p-value ≤0.05.
