# Supplementary material for: Aromatic inhibitors derived from ammonia-pretreated lignocellulose hinder bacterial ethanologenesis by activating regulatory circuits controlling inhibitor efflux and detoxification
Source: Front Microbiol. 2014 Aug 13;5:402. doi: 10.3389/fmicb.2014.00402 (PMC4132294; doi:10.3389/fmicb.2014.00402)
Supplement: Supplementary file 1 [file DataSheet1.ZIP › Table S8.pdf]

Table S8. Levels of LC-derived Inhibitors in ACSH and SynH2 at different stages of growth.

	SynH2 Pre-inoc	SynH2 Exp	SynH2 Tran	SynH2 Stat	ACSH Pre-inoc	ACSH Exp	ACSH Tran	ACSH Stat
**Phenolic Amides and Acids**								
Coumaroyl amide (mM)	1.78 ± 0.06	1.61 ± 0.01	1.78 ± 0.16	1.71 ± 0.31	2.90 ± 0.06	2.74 ± 0.02	2.71± 0.02	2.78 ± 0.35
Feruoyl amide (mM)	1.65 ± 0.04	1.51 ± 0.03	1.74± 0.09	1.66± 0.18	1.24 ± 0.07	1.23 ± 0.05	1.20 ± 0.07	1.21 ± 0.04
Coumaric acid (mM)	0.63 ± 0.02	0.63 ± 0.05	0.67 ± 0.01	0.69 ± 0.04	0.45 ± 0.01	0.47 ± 0.02	0.49 ± 0.03	0.48 ± 0.01
Ferulic acid (mM)	0.25 ± 0.01	0.25 ± 0.02	0.26± 0.01	0.26 ± 0.04	0.054 ± 0.005	0.055 ± 0.002	0.056 ± 0.007	0.056 ± 0.002
**Phenolic and Furan Aldehydes**								
HMF (mM)	0.54 ± 0.19	0.18 ± 0.01	ND	ND	ND	ND	ND	ND
Furfural (μM)	ND	ND	ND	ND	18.59 ± 0.10	1.42 ± 1.42	0.41 ± 0.41	0.3 ± 0.3
**Phenolic and Furan Alcohols**								
BisHMF (mM)	ND	0.66 ± 0.03	1.07 ± 0.1	0.95 ± 0.17	ND	ND	ND	ND
Furfuryl alcohol (μM)	ND	ND	ND	ND	6.37 ± 0.19	17.19 ± 1.16	17.69 ± 0.07	16.67 ± 0.17
**Aliphatic aldehyde**								
Acetaldehyde (mM)	0.04 ± 0.01	1.3 ± 1.7	6.4 ± 0.2	4.6 ± 2.8	0.26 ± 0.04	6.47 ± 0.2	4.9 ± 1.7	5.4 ± 1.8

ND= Below limit of detection.
